# First-in-man study of the PSMA Minibody IR800-IAB2M for molecularly targeted intraoperative fluorescence guidance during radical prostatectomy

**DOI:** 10.1007/s00259-024-06713-x

**Published:** 2024-06-10

**Authors:** Freddie C. Hamdy, Alastair D. Lamb, Iain D. C. Tullis, Clare Verrill, Ines Rombach, Srinivasa R. Rao, Richard Colling, Paul R. Barber, Davide Volpi, Luis Barbera-Martin, J Francisco Lopez, Altan Omer, Aimi Hewitt, Shelagh Lovell, Jane Niederer, Adam Lambert, Joke Snoeck, Claire Thomson, Tom Leslie, Richard J. Bryant, Alessandro Mascioni, Fang Jia, Michael Torgov, Ian Wilson, Jean Gudas, Anna M. Wu, Tove Olafsen, Borivoj Vojnovic

**Affiliations:** 1 Present Address: Nuffield Department of Surgical Sciences, University of Oxford, Old Road Campus Research Building, Headington, Oxford, OX3 7DQ UK; 2 Oxford University Hospitals NHS Trust, Oxford, UK; 3 Department of Oncology, University of Oxford, Oxford, UK; 4 Oxford Clinical Trials Research Unit and Centre for Statistics in Medicine, Nuffield Department of Orthopaedics, Rheumatology and Musculoskeletal Sciences, University of Oxford, Oxford, UK; 5 Comprehensive Cancer Centre, School of Cancer & Pharmaceutical Sciences, King’s College London, London, UK; 6 ImaginAb, Inc, Inglewood, CA USA; 7 Beckman Research Institute, City of Hope, Duarte, CA 91010 USA; 8 University of Sheffield, School of Medicine and Population Health, Sheffield, UK

**Keywords:** Prostate Cancer, Intraoperative imaging, Fluorescence Molecular Imaging, Optical imaging

## Abstract

**Purpose:**

Prostate-specific membrane antigen (PSMA) is increasingly used to image prostate cancer in clinical practice. We sought to develop and test a humanised PSMA minibody IAB2M conjugated to the fluorophore IRDye 800CW-NHS ester in men undergoing robot-assisted laparoscopic radical prostatectomy (RARP) to image prostate cancer cells during surgery.

**Methods:**

The minibody was evaluated pre-clinically using PSMA positive/negative xenograft models, following which 23 men undergoing RARP between 2018 and 2020 received between 2.5 mg and 20 mg of IR800-IAB2M intravenously, at intervals between 24 h and 17 days prior to surgery. At every step of the procedure, the prostate, pelvic lymph node chains and extra-prostatic surrounding tissue were imaged with a dual Near-infrared (NIR) and white light optical platform for fluorescence in vivo and ex vivo. Histopathological evaluation of intraoperative and postoperative microscopic fluorescence imaging was undertaken for verification.

**Results:**

Twenty-three patients were evaluated to optimise both the dose of the reagent and the interval between injection and surgery and secure the best possible specificity of fluorescence images. Six cases are presented in detail as exemplars. Overall sensitivity and specificity in detecting non-lymph-node extra-prostatic cancer tissue were 100% and 65%, and 64% and 64% respectively for lymph node positivity. There were no side-effects associated with administration of the reagent.

**Conclusion:**

Intraoperative imaging of prostate cancer tissue is feasible and safe using IR800-IAB2M. Further evaluation is underway to assess the benefit of using the technique in improving completion of surgical excision during RARP.

**Registration:**

ISCRCTN10046036: https://www.isrctn.com/ISRCTN10046036.

**Supplementary information:**

The online version contains supplementary material available at 10.1007/s00259-024-06713-x.

## Background

Prostate cancer (PC) is the second most common male malignancy globally with 1,414,259 new cases reported in 2021, and 375,304 men dying of the disease [1]. In the US alone, it is estimated that 299,010 new cases will be diagnosed in 2024, with 35,250 prostate cancer deaths [2]. Robot-assisted laparoscopic radical prostatectomy (RARP) is one of the main treatment options for clinically localised PC and represents an evolution from the open anatomical surgical approach developed by Walsh in the 1980s [3]. With the realisation that low-grade low-risk prostate cancer can be managed safely with active surveillance protocols [4], there has been a gradual shift of practice towards surgical treatment of high volume, high-risk and locally advanced disease [5] with variable outcomes. The commonly defined ‘Trifecta’ for optimal outcomes after radical surgery consisting of ‘undetectable post-operative prostate specific antigen measurements, urinary continence and erectile function’ continues to be a challenge to achieve. Despite improved diagnostic imaging with multiparametric MRI, approximately one-third of patients receiving surgery are upstaged to more advanced disease, frequently requiring adjuvant or salvage therapies [6, 7]. The limitations are caused by the inability of surgeons to identify the extent of prostate cancer intraoperatively in order to decide whether the neurovascular bundles situated in the postero-lateral aspect of the prostate gland can be safely preserved or sacrificed, and a further inability to visualise small-volume disease infiltrating lymph nodes. These shortfalls have driven much innovation using prostate-specific membrane antigen (PSMA) in image-guided surgical technologies.

PSMA is a cell-surface protein over-expressed in the majority of prostate cancer cells and represents an ideal target to develop sensitive radio-active and fluorescent tracers [8–10]. The recent establishment of Gallium 68 PSMA-11 ligand PET-CT scanning has transformed the management of recurrent prostate cancer through the identification of small volume extra-prostatic disease and is being evaluated further for pre-operative staging and novel theranostics using labelling with the beta particle emitter Lutetium-177 (^177^Lu) [11].

PSMA is a type-2 integral membrane glycoprotein found in prostate tissues, and is the most well-established, highly restricted prostate-cancer-related cell membrane antigen known, and is expressed by virtually all PCs, as a non-covalent homodimer [12]. In this work, we use a PSMA-targeting minibody. Minibodies distribute relatively quickly in vivo, penetrate target tissues efficiently, and rapidly clear the circulation, making antibody fragments superior to parental antibodies for imaging applications. ImaginAb Inc. (Inglewood, CA, USA) has re-engineered the PSMA J-591 antibody [13] into an optimized targeted imaging agent, known as a minibody [14]. IAB2M is an 80 kDa molecular-weight minibody with an affinity of 0.08 nM that targets the extracellular domain of PSMA, and following conjugation with a NIR dye, forms the basis of the optical imaging agent IR800-IAB2M used in the work reported here.

The IR800-IAB2M tracer was considered safe for clinical use since the minibody had already been used in the clinic [15, 16] and the fluorophore has been used in numerous clinical applications. Similarly, the photo-induced toxicity of the fluorophore, resulting in the production of singlet oxygen, has been shown to be negligible [17].

A variety of approaches have been devised to target PSMA [18]. Small molecules against PSMA have been developed and to date two have been used in man in small pilot clinical studies [19, 20]. Small molecules are attractive because of their short clearance time [21]. In contrast, antibodies exhibit high tissue specificity but due to their high molecular weight, their prolonged binding and clearance times clinical applications can be problematic and impractical. Therefore, the lower molecular weight of minibodies, which exhibit the same specificity benefits of whole-length antibodies, but with shorter clearance times make them highly attractive in image guided molecular targeting, hence the rationale of our work. The Prostate MOlecular Targeting to Enhance surgery (ProMOTE) pilot study presented herein is a first-in-man interventional trial of a specific PSMA minibody tracer (IAB2M) conjugated to a near infra-excited fluorophore for intraoperative imaging and the identification of extra-prostatic malignant tissue in the prostatic neurovascular bundles and regional lymph nodes during RARP. The study used a Fluorescence Molecular Imaging (FMI) approach exploiting an optical platform developed at Oxford to image simultaneously white light and near-infrared (NIR) fluorescence. The results and implications for future use of this technology are discussed.

## Materials and methods

### The IR800-IAB2M tracer

The anti-PSMA minibody (Mb), IAB2M, was conjugated with the NIR dye, IRDye800CW-NHS ester (LI-COR Biotechnology, Lincoln, NE) on lysine residues in 0.1 Na Borate buffer pH 8.5 using ≥ 3 molar excess of dye for 2 h at room temperature to produce the optical imaging agent IR800-IAB2M used here.

This agent was purified and characterised in vitro, as described in Supplementary information (§S1-§S6), and its optimal Fluorophore-to-Minibody ratio (FMR) determined to be ≤ 2. The agent’s binding properties in PSMA-expressing and PSMA-non-expressing cell lines as well as its binding specificity were evaluated, as described in Supplementary information §S5, prior to undertaking in vivo work. A custom microscope platform [22] adapted for work at 785 nm excitation was used for cell imaging.

The quantum yield of the fluorophore is estimated to be 10–15% [23], although variations in tissue pH are known to affect yields [24]. The excitation (peak at 775 nm) and emission (peak at 792 nm) spectra of the fluorophore are presented in the Supplementary information (§S8)*.*

### Pre-clinical tumour establishment and imaging

Six- to 8-week-old male nude (nu/nu) and NOD-SCID (Nonobese diabetic-severe combined immunodeficiency) mice (both from Charles River, Wilmington, MA) were housed and treated according to an approved UCLA’s (University of California Los Angeles) Chancellor’s Animal Research Committee (ARC) protocol.

For tumour establishment, cells were harvested, re-suspended with a 1:1 ratio of Matrigel (BD Biosciences, Bedford, MA) in FBS (foetal bovine serum)-free media and implanted subcutaneously into the shoulder region of the mice. The route of administration of IR800-IAB2M for all studies was intravenous. Eight (*n* = 8) male nude mice were injected via the tail vein with 100 µg of IR800-IAB2M in a volume of 200 µL. At 48 h, organs were collected from 4 of the mice and imaged ex vivo. The remaining 4 mice were imaged in vivo at 72 h. The organs were harvested and imaged ex vivo at the time of sacrifice. To show specificity, additional nude mice were engrafted with CWR22Rv1 in the right shoulder and PC3 (PSMA negative cells) in the left shoulder. These mice were imaged with in-house-developed NIR imaging device at different times after dosing with 50 µg of IR800-IAB2M with an FMR of 1.2).

### Clinical evaluation

#### Patients

Twenty-three male participants with prostate cancer diagnosed through Urology clinics at Oxford were enrolled after obtaining informed consent. The study was registered (ISRCTN10046036) and approved by the Ethics Committee (REC No. 18/SC/0103). Inclusion criteria were histologically proven non-metastatic localized or locally advanced (cT3) PC using parameters of unfavourable disease: PSA ≥ 10 ng/ml and biopsy ISUP grade 3 (Gleason score 4 + 3 = 7), or ISUP grade 4–5 or clinical stage equal to or higher than T2c. Between July 2018 and January 2020, the patients received a robot-assisted laparoscopic radical prostatectomy and extended pelvic lymphadenectomy (ePLND) using the Da Vinci® surgical robot and stereoscopic white-light reflectance imaging. Table 1 provides patient baseline clinical characteristics. Patients were administered doses of IR800-IAB2M intravenously ranging between 2.5 mg-20 mg, at intervals ranging between two days and seventeen days before surgery.

#### Surgical technique

Intraoperative imaging during RARP was used with the FMI technique to identify extra-prostatic tumour. A handheld laparoscope was inserted through a 12 mm assistant-port. This provided near-real-time fluorescence images simultaneously with image-guidance afforded by white-light reflectance to identify tumour lying outside the prostate. Following patient positioning, routine insertion of the laparoscopic ports and docking of the robot, fluorescence imaging was applied to check for any areas of fluorescence along the lymph-node chains from the obturator fossa to the common iliac vessels. An ePLND was performed. Fluorescent areas were sampled for histopathological examination. RARP technique varied according to the surgeon, including both standard anterior and posterior (Montsouris) approaches. Continence and erectile function preserving elements were included as indicated. Fluorescence imaging was used at several stages throughout the procedure. Fluorescence was applied: (a) prior to the bladder taken down to visualise the pelvic lymph-nodes, before, during and after ePLND; (b) immediately after the bladder was taken down; (c) after dissection of the prostate and neurovascular bundle (NVB) if appropriate and prior to transection of the urethra, to identify any fluorescing areas outside the boundary of the prostate, including the NVB, apex and bladder-neck; and (d) following transection of the urethra and removal of the prostate with the seminal vesicles.

### The fluorescence imaging platforms

#### Pre-clinical imaging

The in vivo preclinical evaluation of IR800-IAB2M was performed at the Preclinical Imaging Technology Center (PITC) of The Crump Institute for Molecular Imaging (UCLA, USA). A NIR imaging device [25, 26] developed in Oxford was used; this device is uniquely sensitive and capable of providing fluorescence images in near real-time simultaneously with conventional white light reflectance, by selectively preventing the fluorescence excitation light from reaching the imager. Fluorescence excitation was at 785 nm and the emission was collected over 820–900 nm. This arrangement was compatible with fluorophore excitation and emission spectra, presented in Supplementary Information (§S8), along with technical details of the in-house pre-clinical imager (§S7.1.)

#### Clinical imaging

For the clinical study, an in-house developed imaging device was used in conjunction with an achromatic electrically focus-programmable lens [27], and a NIR-transmitting laparoscope type 26003AGA, (Karl Storz, Tuttlingen, Germany) without any filters selected on the laparoscope. A custom notch filter (Alluxa Inc, Santa Rosa, CA, USA) with a very deep notch (OD 9) at 780 nm was used after the laparoscope output and before the programmable lens to provide rejection of laser excitation while allowing both white light (colour) and fluorescence imaging at > 795 nm to be performed. Technical details of the clinical imager are presented in the Supplementary information (§S7.2.). This system provided a wide programmable dynamic range of imager sensitivity (up to 16x) and integration times (up to 1 s). In addition, the camera permits pixel binning that can be used to further increase sensitivity at the expense of spatial resolution.

We use the standard gamma compression (γ = 0.45) as defined international Rec.709 standard [28]. Rec.709 specifies a slope of 4.5 below a relative luminance value of 0.018, and scales and offsets the pure power function segment of the curve to maintain function and tangent continuity at the breakpoint.

### Clinical image analysis

On playback with Adobe Premier Pro (Adobe, San Jose, CA, USA), segments of video were selected to match specimens as described in the histology reports. Each frame of video is captured as a Portable Network Graphic file (.png) [29]. Relevant image frames were selected where motion artefacts were negligible and where a portion of tissue with a similar working distance could be used to provide background intensity information. ImageJ 1.53f [30] is used to make masks (using ‘selection’ and ‘generate mask’ tools) and appropriate regions of tissue and background were selected. An attempt was made to keep these regions to cover approximately the same number of pixels. Since working distance could not be readily controlled, and hence excitation intensity could not be controlled, the signal-to-noise ratios in selected regions across all images were inevitably different but the number of pixels used was large enough for this not to cause significant errors in determining the tumour to background ratio.

Using a Matlab R2019b [31] script, images were converted to a floating point array, de-gamma'd and black level corrected; all subsequent measurements were made on these corrected, linear intensity scale images. Black level correction was made using previously acquired ‘blank’ images, with black levels acquired at all camera gains, integration times, and binning. Two masks were applied, one corresponding to the bright region (or regions) of the image, the other to a nearby background region (or regions). These regions corresponded to nominal tumour and normal tissue, assuming marker specificity. The mean values of the two sets of masked pixel values were calculated. The ratio of the mean of tumour pixels to mean of background pixels was calculated and used to present the tumour to background (TBR) data reported here.

### Clinical tissue sample preparation and imaging

Formalin-fixed, paraffin-embedded specimens were processed and evaluated for tumour presence according to ISUP (International Society of Urological Pathology) and RCPath (Royal College of Pathologists) protocols [32, 33] by a board-certified uropathologist, using a digital pathology system (Philips Electronics, Eindhoven, The Netherlands) from 4 µm thick conventional diagnostic H&E sections. Consecutive sections were further evaluated as follows: one slide for PSMA expression and a 10 μm thick slide for evaluation of tissue fluorescence.

#### PSMA immunohistochemistry

Formalin-fixed paraffin-embedded sections were heated to 60 °C for 10 min, deparaffinised in xylene and rehydrated in graded concentrations of ethanol. Endogenous peroxidase was neutralised with 3% H_2_O_2_ in methanol for 10 min at room temperature. Antigen was retrieved using citrate buffer pH6 for 10 min at 95 °C and samples were left to cool in the buffer for 20 min. Samples were blocked with PBS/5% NGS for 30 min at room temperature. Mouse PSMA monoclonal antibody (GTX19071 Gene Tex, Irvine, California USA) diluted 1/100 in PBS/5% NGS was applied to the samples overnight at 4 °C. The sections were then incubated with Biotinylated goat anti mouse IgG (BA-9200, Vectorlabs, Kirtlington, United Kingdom) diluted 1/250 in PBS/5% NGS for 30 min at room temperature. The detection system was ABC reagent (Vectorlabs PK-7100) and DAB Substrate Kit SK-4100 (Vectorlabs). The counterstain was Harris’s haematoxylin.

#### Clinical tissue fluorescence imaging

Fluorescence emission from surgically excised tissue slices was imaged with an Odyssey CLx (LI-COR Biosciences Ltd., Milton, Cambs, UK) with Image Studio Version 5.2.5 software at a spatial resolution of 21 μm, using excitation wavelengths of 685 nm and 785 nm and emission wavelength bands of 710–730 nm and 812–832 nm respectively. Images acquired only at 785 nm excitation are presented here, while the 685 nm excitation was useful to identify the complete sections, for alignment purposes.

### Measurement of marker excretion in urine

Urine samples were collected twice a day (morning and evening) by patients in pots and labelled with study number, date and time and refrigerated. Time zero was taken as the time of administration of the marker and collection continued for a maximum of 17 days post administration (see SF8 in Supplementary Methods). The samples were decanted into 2.0 ml Eppendorf (Eppendorf Safe-Lock Tubes, 2.0 mL, colourless polypropylene 0030120094) and centrifuged (for 10 min) and the Eppendorf contents decanted into a fresh 2.0 mL Eppendorf. These tubes were loaded into black Delrin® (acetal) block with window 10 mm × 5 mm and imaged with our in-house pre-clinical system at a range of 210 mm and fluorescence excitation at 780 nm. The camera settings were either 500 ms integration or 160 ms integration (if saturated) and at × 4 gain. Once again images were converted to greyscale images using the Rec.709 algorithm in order to derive intensity information on a linear intensity scale. A mask was used to remove any ‘dead’ pixels. The mean values and the standard deviation of the pixel intensity values in each sample image were determined. Although we did not correct for potential changes in fluorescence excitation power, past experience indicates that this is  < 1% and far lower than the observed inter- or intra-patient variability in fluorescence intensity.

### Analysis plan

We used the ability of surgeon-perceived contrast threshold as the indicator of potential tumour tissue to determine sensitivity (proportion of tissue samples for which intraoperative fluorescence correctly identified cancer seen on histology) and specificity (proportion of tissue samples for which intraoperative fluorescence correctly excludes the presence of cancer, as confirmed by histology). Sensitivity and specificity were generated for each participant separately for lymph nodes and extra-prostatic tissue. Surgeon-perceived contrast thresholds were used as we had not developed fully intraoperative capabilities for real-time measurements of TBRs.

## Results

### IR800-IAB2M localizes to PSMA expressing xenografts

**Fig. 1:**
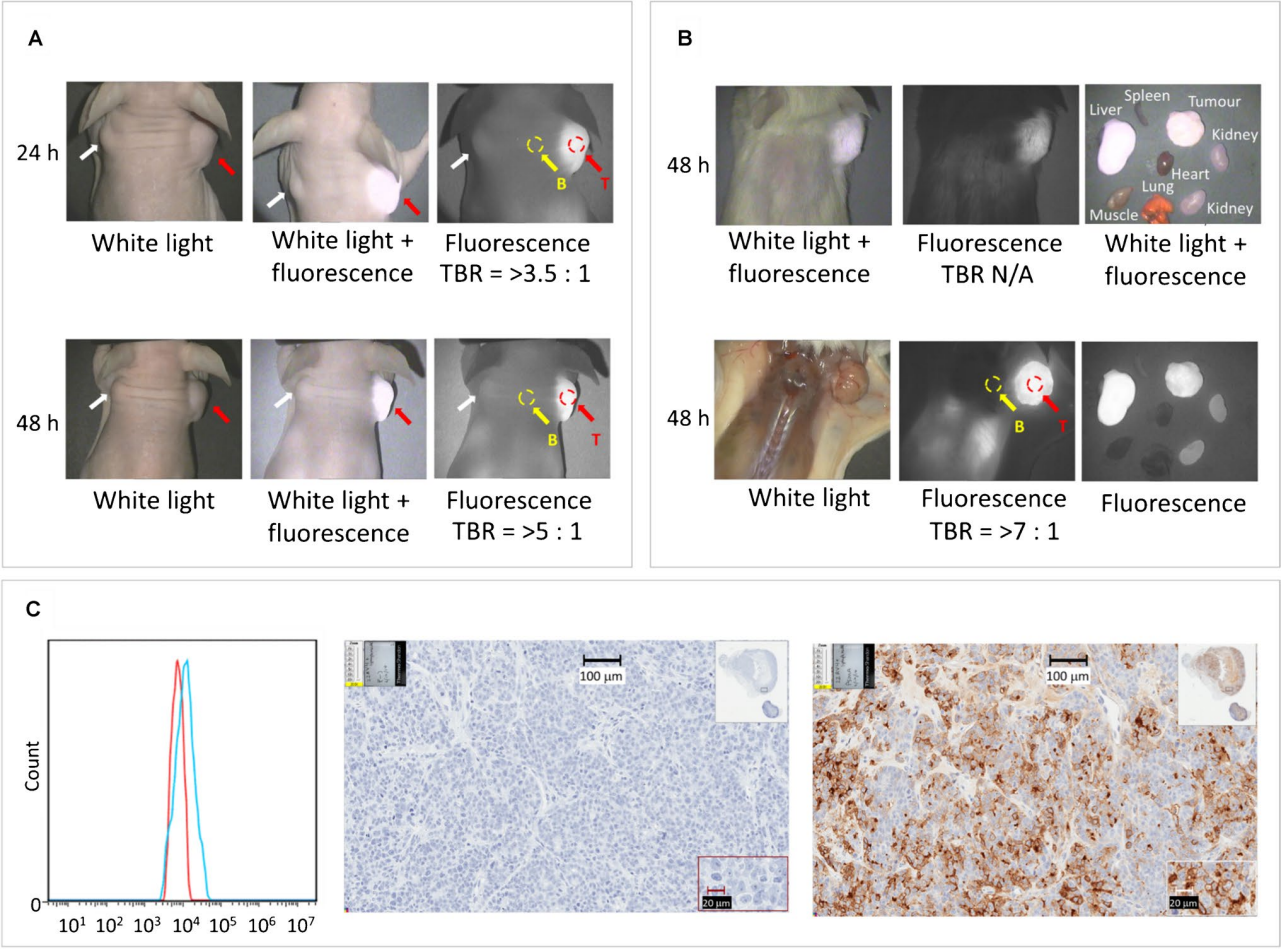
Optical imaging with pre-clinical NIR Imaging camera and IHC. Panel **A**: White light and fluorescence images of nude mice bearing human CWR22Rv1 (red arrow) and PC3 (white arrow) prostate cancer xenografts at 24 and 48 h post injection of IR800-IAB2M; approximate tumour to background ratios (TBR) are determined from tumour (red circle) and normal (yellow circles) tissue regions. Panel **B**: White light and fluorescence images of SCID mouse bearing human CWR22Rv1 xenografts and harvested tissues at 48 h post injection of IR800-IAB2M. TBRs defined as in panel A. Panel **C**: PSMA expression on CWR22Rv1 cell lines and in tumour xenografts confirmed by FACS (left) and IHC (right)

The specificity of the probe was evaluated after bilateral implantation of additional nude mice with CWR22Rv1 in the right shoulder and PC3 (PSMA negative cells) in the left shoulder using the Oxford NIR camera (Fig. 1A). Good tumour contrast was observed one day post injection for CWR22Rv1 tumours. Low background level of fluorescent signal was observed in the PSMA negative PC3 tumours (*n* = 3) likely due to the enhanced sensitivity of the camera. Image contrast was further improved when animals were imaged two days post injection of IR800-IAB2M. In addition, NOD SCID mice with CWR22Rv1 tumours were also imaged (Fig. 1B). In both animal strains, the fluorescence signal from the CWR22Rv1 tumour was clearly visible through the skin with limited background in the rest of the mouse. Post-mortem images revealed that the fluorescent signal was limited to the tumour, liver and kidneys in the same mouse. The expression of PSMA on CWR22Rv1 cell line and in tumour xenografts was confirmed by FACS analysis and immunohistochemistry (Fig. 1C). Results of additional pre-clinical imaging are presented in Supplementary information §S6.

### Clinical imaging

Between July 2018 and January 2020, 23 men with prostate cancer scheduled to receive a RARP gave their informed consent to participate in the feasibility trial. Table 1 summarises their clinicopathological baseline data. At the time of surgery, clearly delineated fluorescing regions were excised and sampled for histopathological evaluation and microscopic fluorescence. Six of the 23 patients are presented individually in more detail as exemplars and are summarised below. As this was a first-in-man investigation, full comprehensive evaluation was not possible in all men because of a combination of factors, including short interval between administration and surgery causing poor fluorescence contrast, learning curve to minimise and control laparoscope tip-to-tissue working distance, over-emphasis on nodal disease in men with low probability of lymph node involvement rather than surgical margins in early cases, and technical issues with instrumentation. Although the manufacturer (ImaginAb) recommendation was to use a starting dose of 20 mg/patient intravenously prior to surgery, we have had sufficient sensitivity to use lower doses, down to 2.5 mg, and different intervals between injection and surgery ranging between 1 and 17 days. In most cases, intervals of 5–10 days appeared to provide adequate marker clearance, while longer intervals did not improve contrast, and non-specific fluorescence signals were excessive at shorter intervals.

### Analysis of clinical images

A summary of relevant findings in the full patient cohort is presented in Table 1. Intraoperative fluorescence intensity as well as contrast between labelled and surrounding tissues were both reduced and more heterogeneous than observed during pre-clinical work. We relied on surgeon-perceived contrast threshold as the indicator of potential tumour tissue as we did not have access to intraoperative real-time determinations of TBRs. Such a ‘binary’ measure was compared with pathology results to generate the statistical measures reported in Table 1. Although TBRs could be determined retrospectively, a receiver operating characteristic (ROC) curve [34] that includes all doses and times could not be generated reliably, nor could a time-dependent ROC curve [35] be generated due to restricted data availability. Nevertheless, in the cases that were fully evaluable, fluorescence contrast and hence TBRs were determined and compared with standard histopathological analysis. TBRs of 1.3:1 or higher were indicative of a high probability of cancer cell presence in the tissue imaged.

Cases used as exemplars are highlighted in Table 1 and imaging data are presented below for these cases. When tissue samples which showed intraoperative fluorescence were examined histologically, and aligned with perceived fluorescence measures, the overall sensitivity and specificity in detecting non-lymph-node extra-prostatic cancer tissue were 100% and 65% respectively, and 64% and 64% respectively for lymph node positivity. There were no side-effects associated with injection of the reagent.

**Table 1:** Details of patients in first-in-man study

											Intraoperative fluorescence			
											Sensitivity		Specificity	
Patient ID	Dose (mg)	Interval (Days)	Weight (kg)	PSA (ng/ml)	Clinical TNM stage	mpMRI PI-RADS	Biopsy ISUP grade	Pathological stage	RP ISUP grade	Surgical Margin	Lymph Nodes	Extra-prostatic tissue	Lymph Nodes	Extra-prostatic tissue
1	20	1	84	84.19	T2cN0M0	5 Bilateral	5	T3bN1M0	5	+ ve	100%	n/a	60%	50%
2	20	2	82	36.8	T3aN0M0	5 Bilateral	4	T3bN1M0	5	+ ve	100%	n/a	50%	50%
3	20	2	97.3	12.3	T3aN0M0	4 left PZ	5	T3bN1M0	5	+ ve	50%	n/a	80%	67%
4	20	2	78.1	8.80	T2aN0M0	2 right PZ	4	T3bN1M0	3	–ve	n/a	n/a	100%	50%
5	20	2	75	26.16	T2cN0M0	5 bilateral	4	T3bN0M0	2	+ ve	n/a	n/a	63%	100%
6	20	3	84	10.72	T2bN0M0	5 left PZ	4	T2bN0M0	5	–ve	n/a	n/a	17%	100%
7	10	2	102.8	11.30	T2aN0M0	3 right PZ	5	T3aN0M0	5	+ ve	n/a	n/a	67%	100%
8	2.5	3	68	6.50	T3bN0M0	nil	5	T3bN1M0	5	+ ve	33%	n/a	100%	0%
9	2.5	5	91.5	17.64	T2cN0M0	5 left PZ	2	T3aN0M0	2	+ ve	0%	n/a	67%	33%
10	5	4	87.4	5.08	T3bN1M0	5 right PZ	3	T3bN1M0	3	+ ve	83%	-	75%	-
11	5	5	84	33.25	T2bN0M0	5 right PZ	4	T3aN0M0	3	+ ve	n/a	n/a	63%	67%
12*	5	5	80	13.40	T2bN0M0	5 right PZ/apex	4	T3aN0M0	3	+ ve	0%	n/a	71%	100%
13	10	5	120	46.37	T2bN0M0	4–5 bilateral	3	T3bN1M0	3	–ve	50%	-	50%	-
14	5	5	84.6	7.98	T2cM0N0	5 left PZ	3	T3bN0M0	3	–ve	n/a	-	60%	-
15*	5	4	82.8	6.83	T3aN1M0	3–5 bilateral	5	T3aN1M0	5	–ve	100%	n/a	17%	100%
16*	5	7	90.8	12.14	T2bN0M0	5 left PZ	4	T3aN0M0	3	+ ve	n/a	100%	100%	n/a
17	5	7	74.2	6.77	T2bN0M0	4 bilateral	5	T2cN0M0	3	–ve	n/a	n/a	80%	100%
18	10	10	80.1	17.12	T2cN0M0	5 right; 3 left	5	T3aN1M0	3	–ve	0%	n/a	100%	100%
19*	10	10	96.3	10.48	T3bN0M0	5 midline	3	T3bN0M0	3	–ve	n/a	n/a	100%	100%
20	20	10	71.5	19.23	T3aN0M0	5 left PZ	4	T3aN1M0	4	–ve	100%	n/a	78%	67%
21*	20	10	87.5	23.9	T2cN0M0	3 midline	4	T3bN0M0	3	+ ve	n/a	100%	63%	33%
22	20	17	73	12.4	T3bN0M0	5 bilateral	2	T3aN0M0	3	–ve	n/a	100%	67%	67%
23*	20	17	72.1	62.4	T2aN0M0	2 left anterior	2	T3aN0M0	3	+ ve	n/a	100%	50%	60%

‘-‘ indicates that no tissues were sampled; the term ‘n/a’ is used when the relevant parameter could not be calculated (i.e. when a division by zero occurred)

*Exemplar cases described in detail below

#### Case 1

**Fig. 2:**
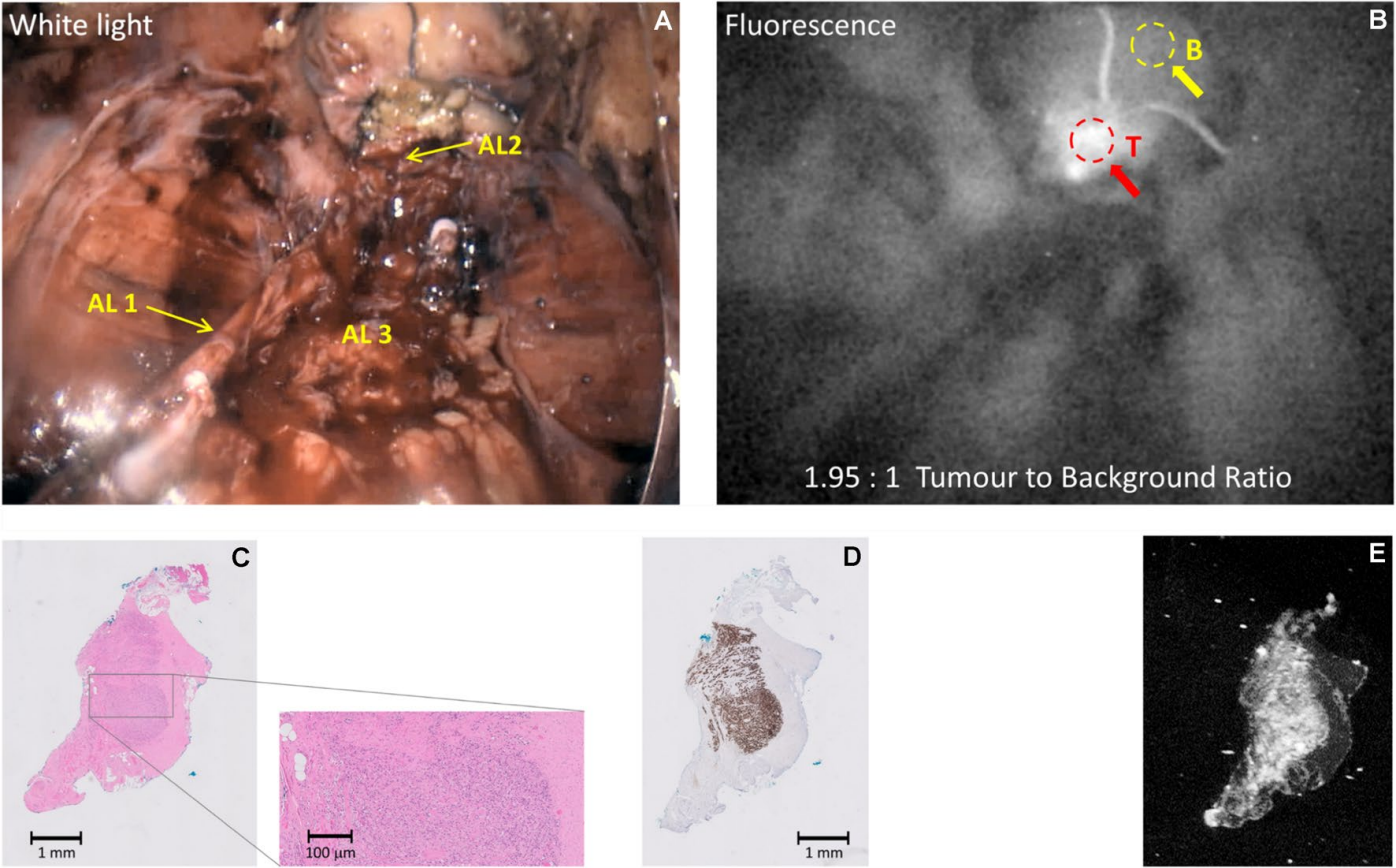
(Dose 5 mg; Interval 120 h) Example of imaging of the dorsal vein complex and anterior reresection margin indicating a positive apical margin fluorescence in patient 12. Panel **A**: White light illumination: AL1: Left neurovascular bundle; AL2: transected urethral edge, apical area of positive fluorescence (tumour present); AL3: prostatic bed (prostate has been removed); Panel **B**: NIR Fluorescence excitation; Panel **C**: H&E (haematoxylin & eosin) stain; Panel **D**: Adjacent section, PSMA IHC stain; E: Adjacent section, tissue NIR Fluorescence

(Patient 12) 70-y old man, PSA 13.4 ng/ml, multiparametric Magnetic Resonance Imaging (mpMRI) showed a PI-RADS 4 (Prostate Imaging-Reporting and Data System) lesion, right apical region of the prostate; and PI-RADS 3 lesion, transition zone. Targeted biopsies: right sided prostatic adenocarcinoma, ISUP grade 4 (Gleason score 4 + 4 = 8) in targeted and systematic biopsies. There was no clinical or pre-operative imaging evidence of extra-prostatic disease. During surgery and after removal of the prostate and transection of the urethra, the resected apical margin showed high levels of fluorescence (TBR = 1.95:1) and was sampled, confirming the presence of tumour (ISUP grade 3, Gleason score 4 + 3 = 7), as illustrated in Fig. 2. The final histopathological stage and grade was pT3aN0, ISUP grade 3 with a positive apical margin. Residual tumour was positive for PSMA immunohistochemistry and microscopic fluorescence.

It is tempting to quantify the co-localisation of the IHC and tissue NIR fluorescence in Fig. 2 (panels D and E) and corresponding panels in the subsequent surgical imaging figures. However, it is unlikely that the IHC and NIR markers will target the same epitope and some cross-reactivity may be present. The adjacent tissue sections will be processed in separate pathways and some folding or damage to tissue slices may occur. Slice rotation and potential flipping is also likely to occur. These factors will inevitably alter the results of any co-localisation measurements. In this study, such co-localisation measures were not computed, but work to optimise the procedures used in this workflow is ongoing.

#### Case 2

**Fig. 3:**
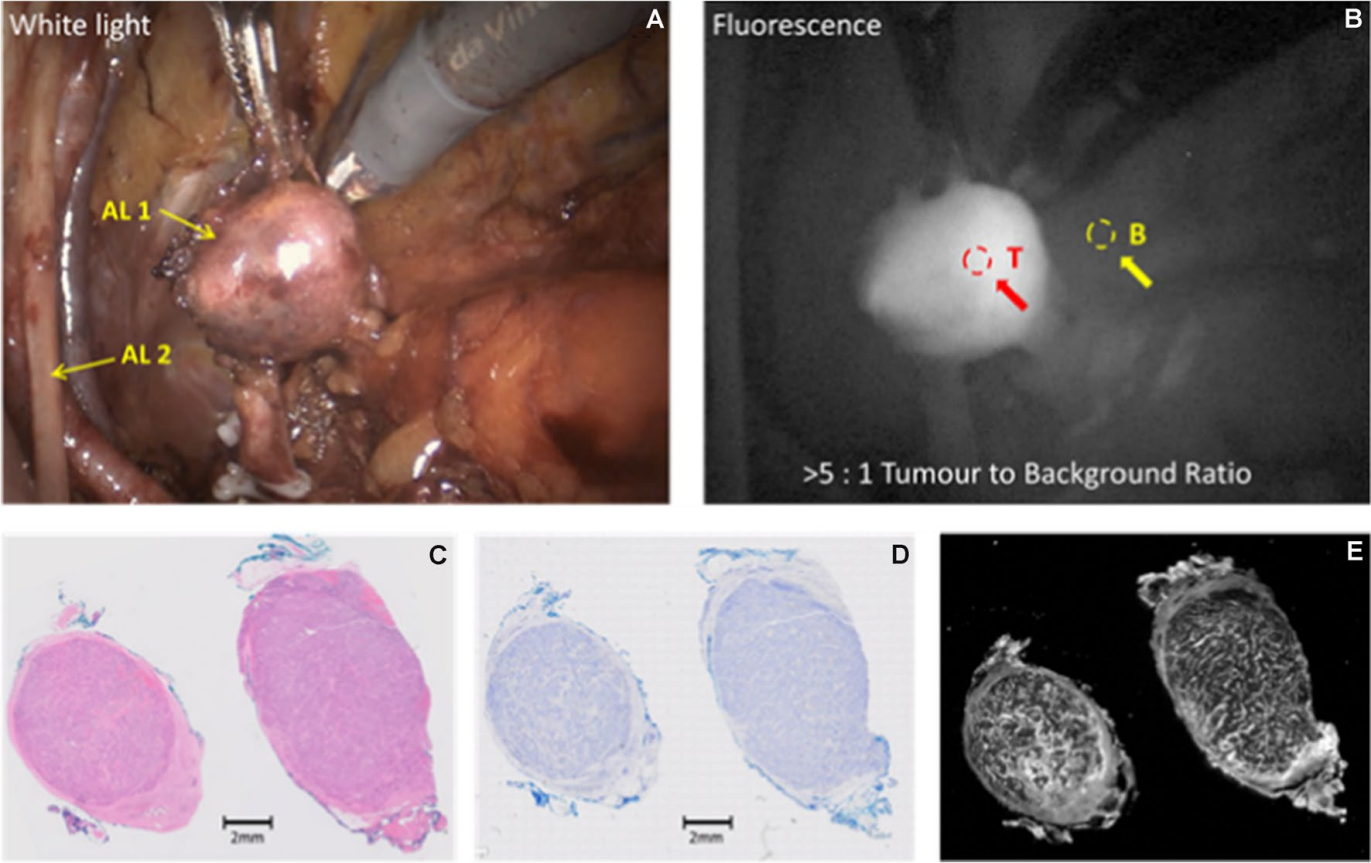
(Dose 5 mg; Interval 96 h) Example of imaging of a large node with positive intraoperative fluorescence in Patient 15. Panel **A**: White light illumination: AL 1: Internal iliac lymph node; AL 2: Left obturator nerve and vessels; Panel **B**: NIR Fluorescence excitation; Panel **C**: H&E stain; Panel **D**: Adjacent section, PSMA IHC stain; E: Adjacent section, tissue NIR Fluorescence

**Fig. 4:**
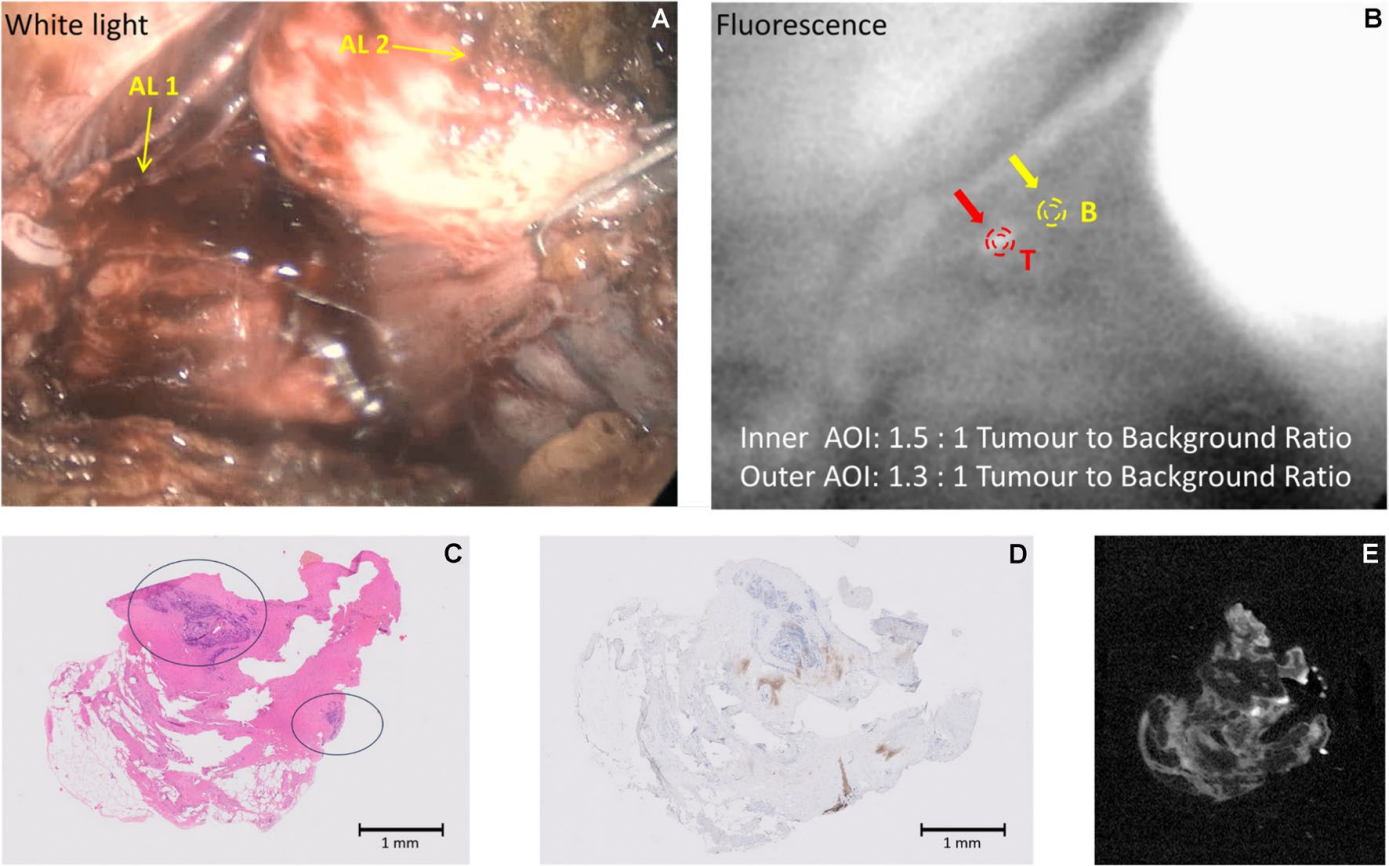
(Dose 5 mg; Interval 168 h) Example of imaging of neurovascular bundle with a positive intraoperative fluorescence in patient 16. Panel **A**: White light illumination: AL 1: Left neurovascular bundle, with fluorescing area (tumour); AL 2: prostate retracted towards the right side and dissected off left neurovascular bundle; Panel **B**: NIR Fluorescence excitation; Panel **C**: H&E stain, with regions of tumour indicated; Panel **D**: Adjacent section, PSMA IHC stain; **E**: Adjacent section, tissue NIR Fluorescence

(Patient 15) 63-y old man, PSA 6.8 ng/ml, mpMRI showed a PI-RADS 5 lesion in the left peripheral zone, a right PIRADS-3 lesion in the right basal region, and a 12 mm left pelvic lymph node in the obturator region. Targeted and systematic biopsies showed left-sided prostatic adenocarcinoma in all 6 cores, ISUP grade 5 (Gleason score 4 + 5 = 9). During surgery, a left sided pelvic internal iliac lymph node was strongly fluorescent (Fig. 4; TBR > 5:1). The final histopathological stage and grade were pT3aN1, ISUP grade 5 with negative surgical margins. The fluorescent lymph node was weakly PSMA positive and strongly fluorescent microscopically. Imaging data are presented in Fig. 3 for this case.

#### Case 3

(Patient 16) 58-y old man, PSA 12.6 ng/ml at diagnosis, mpMRI showed a large left-sided PI-RADS 5 lesion. Biopsies showed bilateral prostatic adenocarcinoma ISUP grade 4 (Gleason score 4 + 4 = 8). No clinical or imaging evidence of extra-prostatic disease. During surgery, and after separation of the neurovascular bundle on the left side, there was an area of fluorescence (TBR = 1.5:1) which was sampled. This is shown in Fig. 4. The final histopathological stage and grade were pT3aN0, and the sampled tissue from the left neurovascular bundle showed a 1.5 mm area of prostatic adenocarcinoma, positive for PSMA immunohistochemistry and for microscopic fluorescence.

#### Case 4

**Fig. 5:**
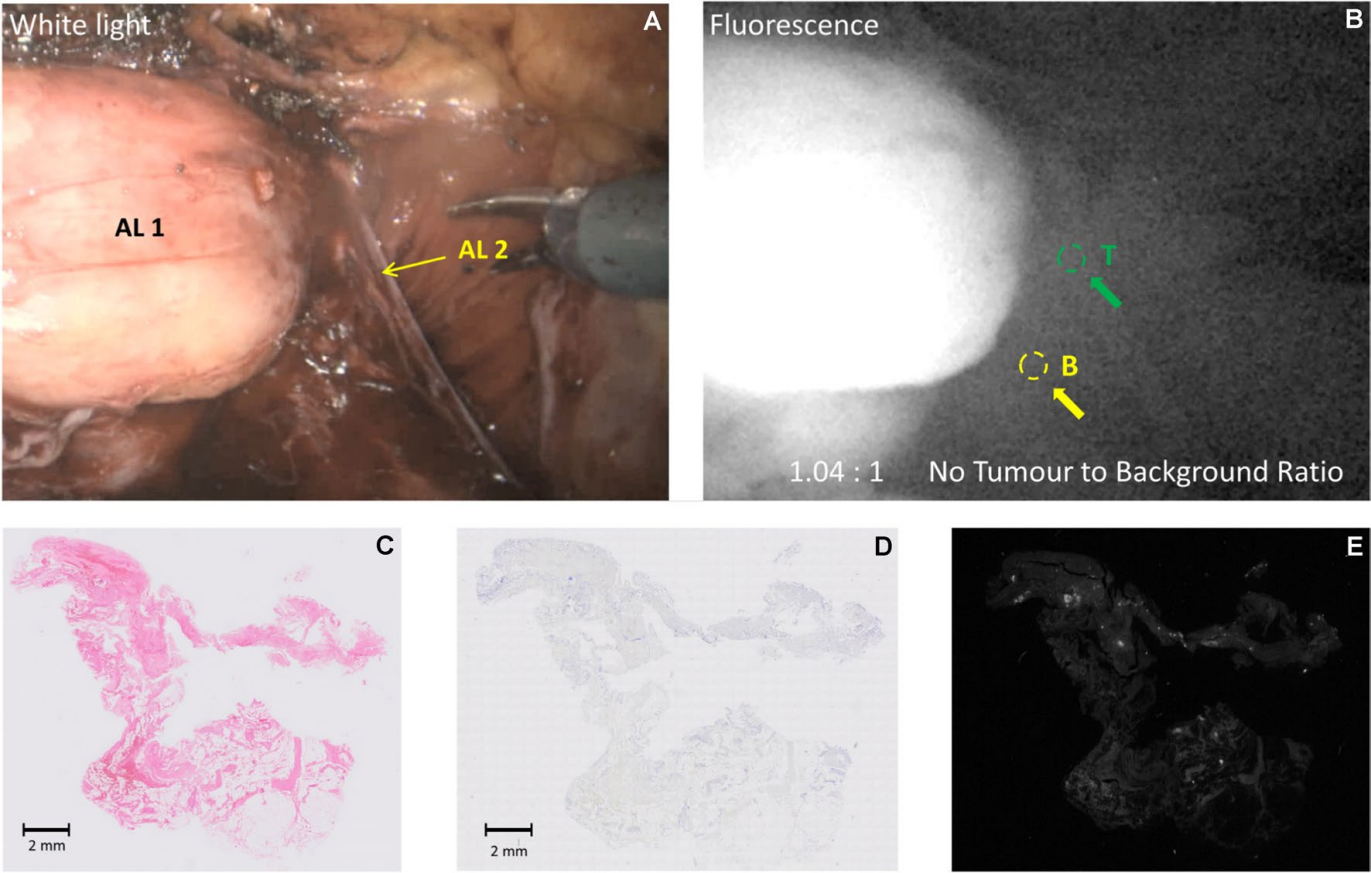
(Dose 10 mg; Interval 240 h) Example of neurovascular bundle with a negative intraoperative fluorescence in Patient 19. Panel **A**: White light illumination: AL 1: Prostate retracted towards the left side; AL 2: Right neurovascular bundle (no tumour, no fluorescence); Panel **B**: NIR Fluorescence excitation; the area depicted with a green “T” shows lack of emission with no tumour present; Panels **C-E** depict adjacent sections from the right neurovascular bundle with no evidence of tumour- Panel **C**: H&E stain; Panel **D**: PSMA IHC stain; Panel **E**: tissue NIR Fluorescence with a faint signal likely due to enhanced detection sensitivity

(Patient 19) 65-y old, PSA 10.4 ng/ml, mpMRI showed a PI-RADS 5 midline lesion extending into the right seminal vesicle. Biopsies showed bilateral prostatic adenocarcinoma ISUP grade 3 (Gleason score 4 + 3 = 7). During surgery, there was an area of fluorescence, (Fig. 5), in the right NVB. The final histopathological stage and grade were pT3bN0, ISUP grade 3, margins negative. This corresponds with the lack of fluorescence in the NVB area images under fluorescence excitation (panel B).

#### Case 5

(Patient 21) 66y-old, PSA 22.3 ng/ml, mpMRI showed extensive PI-RADS 5 lesions bilaterally with early extra-capsular extension left apex and right base. Biopsies showed extensive prostatic adenocarcinoma ISUP grade 4 (Gleason score 4 + 4 = 8). No clinical evidence of other extra-prostatic disease. During surgery, there were two areas of fluorescence (TBRs = 2.2:1 and 1.9:1, Fig. 6), in the right NVB after separation from the prostate which were sampled. The final histopathological stage and grade were T3bN0 ISUP grade 3 (Gleason score 4 + 3 = 7). The sampled fluorescing tissue was positive for PSMA by immunohistochemistry and microscopic fluorescence. Although the TBR in this case is adequate, the fluorescence image suggests that the marker may not have fully cleared from surrounding tissues, due to the high dose administered, despite the long delay time between administration and imaging.

**Fig. 6:**
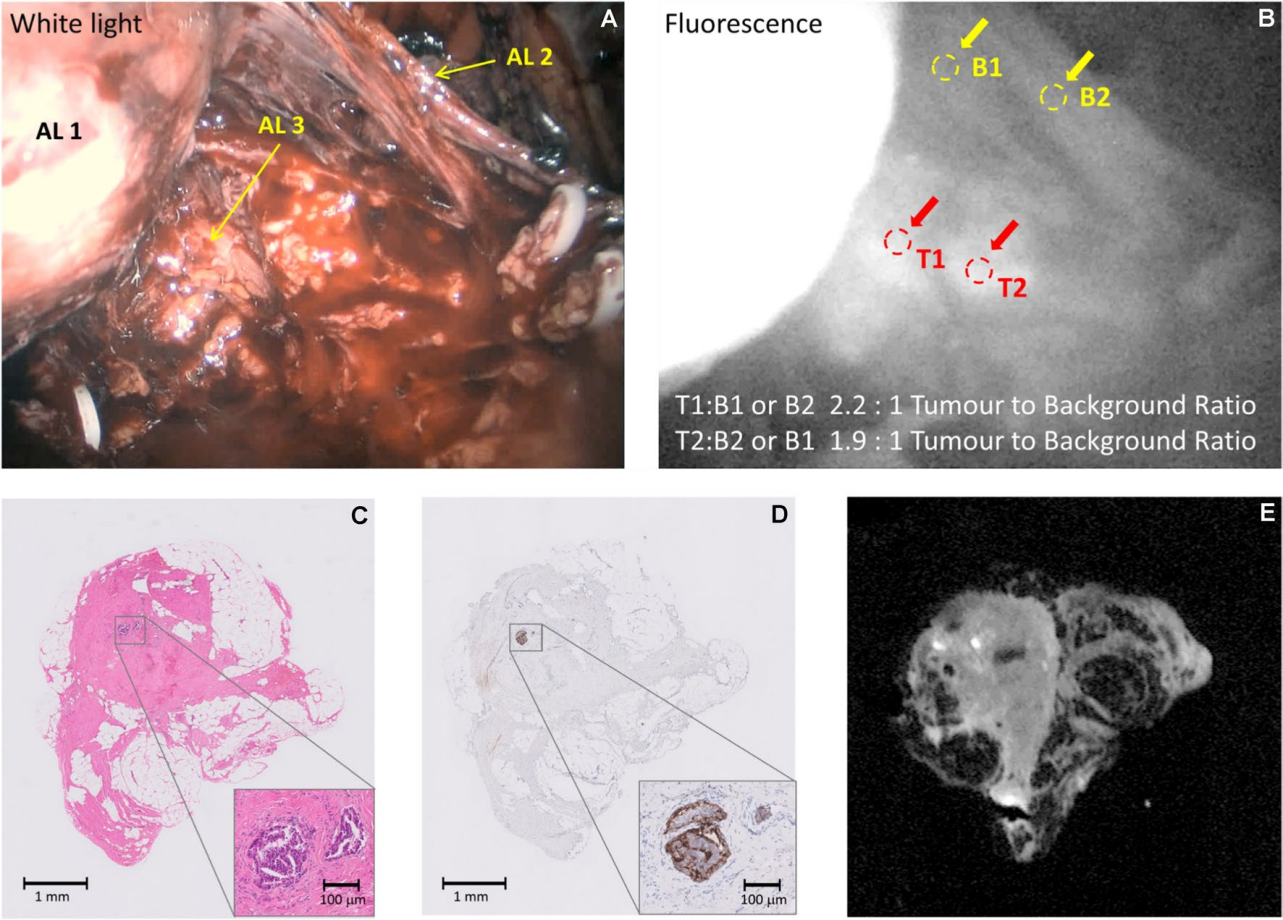
(Dose 20 mg; Interval 240 h) Example of imaging of the extraprostatic tissue showing a positive tumour fluorescence in Patient 21. Panel **A**: White light illumination: AL 1: Prostate retracted towards the left side; AL 2: Right neurovascular bundle dissected off the prostate; AL 3 Areas of fluorescence posteriorly (tumour) between the right neurovascular bundle and the prostate; Panel **B**: NIR Fluorescence excitation; Panel **C**: H&E stain, no tumour; Panel **D**: Adjacent section, PSMA IHC stain, no tumour; **E**: Adjacent section, tissue NIR Fluorescence

#### Case 6

(Patient 23) 71-y old, PSA 46 ng/ml, mpMRI PI-RADS 2 lesion anteriorly left side. Biopsies showed prostatic adenocarcinoma ISUP grade 2 (Gleason score 4 + 3 = 7). No clinical or imaging evidence of extra-prostatic disease. During surgery, and after transection of the urethra, there was an area of strong fluorescence (TBR = 4.8:1, Fig. 7). The final histopathological stage and grade were pT3aN0 and ISUP grade 3 respectively, with a positive apical urethral margin. The sampled fluorescing tissue showed prostatic adenocarcinoma, positive by PSMA immunohistochemistry and microscopic fluorescence.

**Fig. 7:**
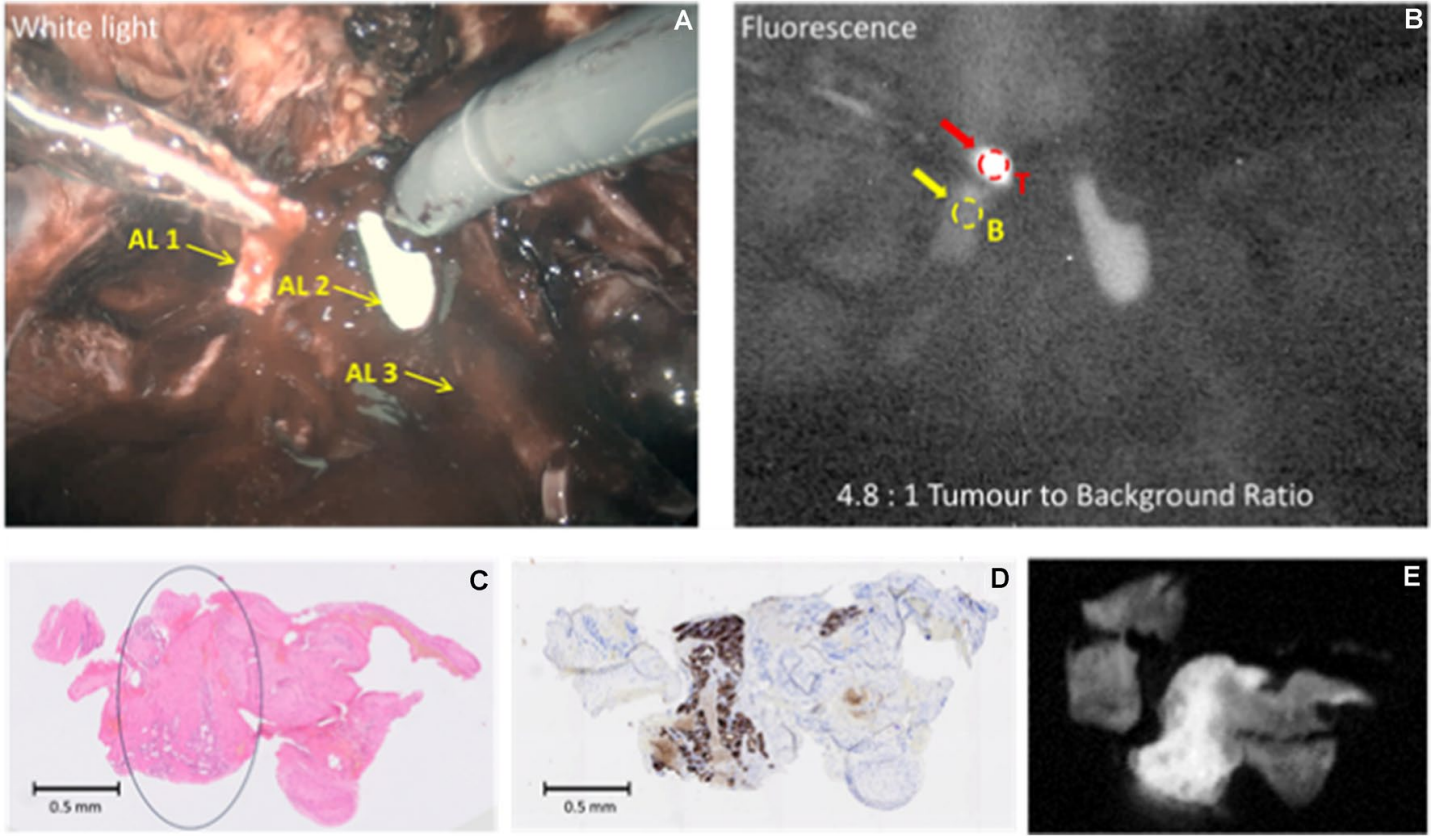
(Dose 20 mg; 408 h) Example of urethral margin with positive fluorescence in Patient 23. Panel **A**: White light illumination: AL1: Posterior apical tissue with fluorescence (tumour present) being excised after removal of the prostate; AL2: Tip of urethral catheter; AL3: Right neurovascular bundle; Panel **B**: NIR Fluorescence excitation; Panel **C**: H&E stain, with a region of tumour indicated; Panel **D**: Adjacent section, PSMA IHC stain; **E**: Adjacent section, tissue NIR Fluorescence

### Urine excretion

**Fig. 8:**
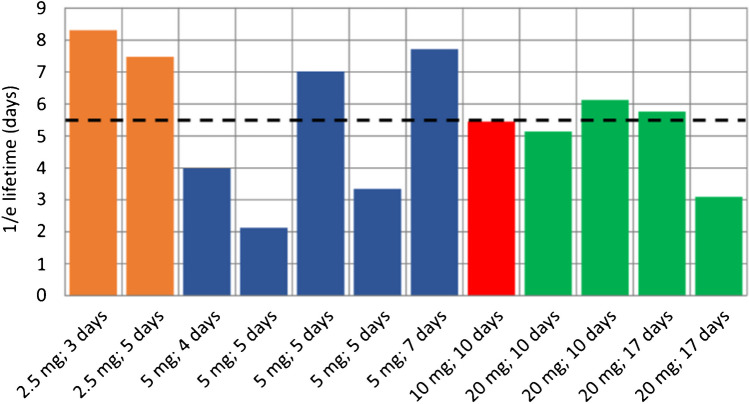
Fluorescence clearance kinetics in urine for different doses and times post marker administration

In an effort to gain an understanding of the marker clearance kinetics and to potentially determine an optimal dose/time combination, the kinetics of loss of fluorescence from urine as a function of time were determined in 12 out of the 23 participants. Even though there was considerable patient-to-patient variability, the clearance 1/e lifetimes were comparable across the cohort, as shown in Fig. 8. Exponential decays were fitted to the absolute fluorescence intensity data from urine from each patient (Supplementary §9 and Figure S8B). A good correlation was found, as expected, with extrapolated intensity at time zero and administered dose (Supplementary Figure S8A). In Fig. 8, the average lifetime is indicated by the dotted line while the different colours identify grouped doses. These data suggest that a significantly greater time should be used between marker administration and surgery compared to that shown in mice.

## Discussion

Over the past four decades, the indications for radical prostatectomy in men with prostate cancer have shifted gradually from low and intermediate localised disease to high-grade, high-volume localised and locally advanced stages. While novel surgical techniques, including the introduction of robot-assisted laparoscopic procedures are used increasingly with good reproducible functional and oncological outcomes, the main challenge remains with the inability of the surgeon to identify reliably extra-prostatic cancer cells during surgery to achieve complete excision of the disease. The use of intraoperative fluorescence during surgery to image tissue of interest is not novel and has relied traditionally on using fluorophores administered through direct injections into organs to demonstrate drainage, or vasculature and facilitate lymphadenectomy and precise excision, using Near Infrared (NIR) imaging to detect fluorescence. The contrast agents used in these approaches are neither tissue nor tumour specific. Fluorescence intraoperative imaging using molecular targets to identify specific tissues was therefore developed using a variety of markers conjugated to fluorophores, and innovations in robotic surgical equipment allow now NIR imaging to enhance precision surgery, with the caveat that NIR and white light imaging has not been achievable to date in real-time during surgery [36].

The increasing use of PSMA as a target for imaging and interventions in prostate cancer is transforming the landscape in managing the disease [11, 23, 24, 37, 38]. For intraoperative FMI of cancer, various tumour-targeting molecules, such as antibodies, nanoparticles, proteins, peptides, and small molecules, have been developed. We report herein the pre-clinical development of a novel minibody against Prostate Membrane-Specific Antigen (PSMA), and its first-in-man use after conjugation with the fluorophore IRDye800CW-NHS ester, coupled with an in-house optical system developed to image simultaneously NIR and white light in an attempt to image extra-prostatic cancer cells during RARP. The rationale for the minibody development was to combine the advantages of targeting specificity associated with antibodies, and the fast clearance kinetics associated with smaller molecules. Preclinical studies suggested a speedy clearance and optimal imaging times of the order of 1–2 days.

Extrapolation of doses and clearance times across species of widely differing metabolic rates is, however, not straightforward [39, 40], particularly when the clearance rate of the marker bound to the prostate tumour tissue was unknown. Although we started with the manufacturer-recommended 20 mg dose, we obtained good clinical results with a dose of 5 mg (equivalent to just over 50 µg/kg) or about twice the level considered to be a micro-dose, [41, 42]. There is considerable interest in using micro-dosing with FMI [43, 44]. With modest improvement in imaging sensitivity, micro-dosing should be achievable for laparoscopic/robot-assisted approaches, as it is in wide-field surgery, i.e. non-laparoscopic imaging [45]. Micro-dosing (phase 0) studies have also been suggested for accelerating clinical translation of novel agents [46] and camera sensitivity plays a critical role here. For example, clinical translation of fluorescently labelled bevacizumab [45], has achieved a signal-to-noise ratio of ~ 4:1 for micro-dosing administration. This performance was obtained using cooled cameras although we achieve a comparable signal-to-noise ratio with an uncooled detector, albeit at long exposure times. Clearly, there is scope for further improvement in camera sensitivity. However, to the best of our knowledge there is currently no definition of ‘sufficient sensitivity’ in clinical fluorescence imaging [47]. Fluorescence imaging sensitivity can be increased by reducing the working distance. This increases the excitation power density according to the inverse square law. Unfortunately, while spatial resolution is enhanced as a result of a reduction of the working distance, the field of view is reduced, making surgical navigation more awkward. Working distance could not be usually reduced significantly with our instrumentation since we were using an assistant port for laparoscopy imaging. FMI demands the use of sensitivity high enough to image the low concentrations typically attained by targeted fluorescence agents, in the range of sub-nM to 100 nM. Camera sensitivity is one of the most critical parameters in reaching high signal-to-noise ratio fluorescence detection in short exposure times. In our case, most of the data reported required exposure times of < 500 ms. FMI sensitivity is also determined by the intensity and spectral response of the fluorescence excitation: here we are forced to limit power densities to below 50 mW/cm^2^ range to eliminate the possibility of tissue heating. Finally, the ability to block excitation light and light from other sources in the detection channel is key; we have used an emission filter with an optical density > 8 at the excitation light wavelength. Furthermore, FMI sensitivity affects the administered dose required. While non-specific, vascular dyes such as indocyanine green (ICG) are often injected in quantities of tens of milligrams when given systemically or ~ 1 mg when given intratumorally [48], reaching concentrations of > 100 nM—2 μM in tissues, targeted agents are used in much lower concentrations [49], since most of the administered dye is cleared from tissues and only a small amount is present in the targeted tissue. Results based on clinical data have shown that due to targeting and clearance, the concentrations of molecularly targeted agents imaged may be five to six orders of magnitude lower than when imaging ICG [48–50]. Furthermore, the camera integration time and sensitivity are fixed, while our custom system allows these to be increased over a wide range. More detailed descriptions can be found in the Supplementary information (§S8 and Figures S6 and S7). Finally, the Firefly applies the fluorescence information as an overlay which seems to be associated with an intensity threshold. Further developments to our system, not described here, permit the real-time readout of TBR information and the existence of intensity thresholding may detract from determining accurate TBR values.

Administration of the marker pre-operatively was well tolerated even at the higher doses and did not generate any specific side-effects. In order to develop measurable consistent metrics to define the level of fluorescence in relation to probability of detecting cancer cells, we sought to quantify the TBR used as a measure of the specificity of the uptake of our imaging agent within the target organ. The presence of imaging agent in the background can be secondary to a low retention rate in the target organ, leading to leakage into surrounding tissues or, as in this instance, to potential preferential uptake by other organs (particularly highly vascular structures such as the liver, spleen, gut and kidneys). In vivo intraoperative fluorescence in each evaluable case was aligned with conventional histopathological assessment and fluorescence microscopy studies as illustrated (Figs. 2–7). In some cases, small amounts of cancer tissue were detected using FMI, which would not have been picked up during a standard procedure. This ability of the surgeon to visualise pathological tissue during surgery highlights the overarching promise of this new technology, irrespective of the reagent or optical system used to achieve precision surgery.

We have shown that our detection device is able to provide reliable indication of low TBRs, down to 1.3. Although TBRs, at least in FMI, much below 2 are considered borderline [51], the use of TBRs of < 1.5 has been reported in the literature [52]. One of the limitations of the approach described here is that TBRs are determined post-operatively and this has highlighted the need to provide real-time indications of TBR from selected small areas of fluorescence. Other limitations included the low sample number, the lack of PSMA PET-CT imaging and the extensive learning curve in applying FMI.

In order to emphasise the practical need for TBRs, we have presented only the raw, fluorescence-only images. Images are linearized prior to TBR determinations. This is in contrast to the more usual approach where overlays, linear or non-linear, with or without threshold or otherwise, or heat maps are applied to the fluorescence data prior to display [53]. The high sensitivity of our system is inevitably associated with somewhat higher noise than when higher marker concentrations are used.

The data presented here can only be considered preliminary but suggest that the IR800-IAB2M is a suitable agent for intraoperative identification of extra-prostatic cancer tissue during RARP. Our surgical robot used in this study did not have the advantage of an integrated system to image NIR fluorescence during surgery and had to rely on our in-house optical system described above. A Phase 3 evaluation using a full randomised design is underway. In this evaluation, provision for real-time, on-line metrics has been incorporated in the imaging device although in the medium term it would be desirable to fully integrate this with robot-assisted equipment that uses the next generation of highly sensitive imagers. One of our findings, particularly at higher doses, is that the number of false positives is higher than would be expected. However, intraoperative fluorescence detection always correlated with fluorescence detected from tissue samples in all samples analysed. Clearly, the false positives are due to either (a) inadequate marker clearance, and/or marker accumulation in slow to clear regions or (b) to inadequate specificity of the minibody, or (c) to low levels of PSMA expression in non-prostatic tissues. The latter is known to occur [54, 55], although this depends on the marker epitope. The sensitivity and specificity of IR800-IAB2M appears to compare favourably with other recent reagents tested such as OTL78 (range of average sensitivity and specificity of 33.3%-68.4%, and 52.6%-100% respectively), and IS-002 (Average sensitivity/specificity of 97% and 45% for lymph nodes, 100% and 80% for residual locoregional disease [22, 23]. The use of lower molecular weight [56] markers may be advantageous, though potentially at the expense of specificity. Comprehensive evaluation was not possible in all men. In the early part of the study, we have focused on imaging the pelvic lymph node chain, and not on extra-prostatic non-lymphatic tissue. Results from lymph-node imaging were variable with inconsistencies in the early evaluations. When it was decided to focus on non-lymphatic tissue, we were allowed by our regulatory approvals to study a limited remaining number of patients which we are reporting in more detail herein.

Marker clearance, at least as measured by urine fluorescence, provides only a ‘global’ indication of clearance, and does not reflect the timescales of localised clearance. In particular, clearance from lymph nodes would be expected to be slow, due to the molecular weight of IR800-IAB2M. The apparent lack of agent elimination even at delayed time points in some cases, as well as spatial resolution can pose challenges in tissue margin delineation. We recognise that this represents a limitation of our pilot work, which we will endeavour to overcome by continuing to refine and validate real-time accurate determination of TBRs in the next phase of our evaluation.

It is informative to explore how other commercially available fluorescence guided devices would perform in comparison with our custom system. Of these the Da Vinci® Xi Firefly^TM^ system is commonly used during robotic procedures. Although we did not have access to this during the procedures described here, such a system has recently become available to us. Unfortunately, the Da Vinci fluorescence system is aimed at ICG imaging and uses an excitation wavelength of 805 nm, well away from the optimal 775 nm for the IRDye800 fluorophore. In addition, the Da Vinci system collects fluorescence light only at >  ~ 830 nm, at which point the IRDye800 fluorescence is no longer significantly emitting. Furthermore, the camera integration time and sensitivity are fixed, while our custom system allows these to be increased over a wide range. More detailed descriptions can be found in the Supplementary information (§S8 and Figures S6 and S7). Finally, the Firefly applies the fluorescence information as an overlay which seems to be associated with an intensity threshold. Further developments to our system, not described here, permit the real-time readout of TBR information and the existence of intensity thresholding may detract from determining accurate TBR values.

The factors outlined above contribute to our system exhibiting a > 25 × higher sensitivity, for IRDye800 imaging, than that afforded by the Firefly. However, more recent versions of the Firefly system, as used by Nguyen et al. [20] and described therein, do provide significantly enhanced sensitivity. Such systems are likely to be suitable for the procedures described here, though this remains to be established. Furthermore, modifications to optimise the excitation and collection wavelengths are technically straightforward. Other non-robotic devices, such as those used in the work described in [19] are also likely to be appropriately sensitive. However, we did not have access to other laparoscopic fluorescence-capable imaging systems.

In several cases, IHC detection did not match with either intraoperatively detected or microscopy detected fluorescence signals. This highlights the practical difficulties of aligning intraoperatively detected areas of fluorescence with histology and IHC areas. We have found it useful, in the Phase 3 evaluation currently underway to maintain laparoscope working distance and to include an object of known dimensions (e.g. a surgical clip) in the field of view of image on which TBRs are determined. This allows a fixed fluorescence imaging sensitivity to be maintained and to ensure that the areas of fluorescence could be measured and compared with histology findings.

In conclusion, our study demonstrated the pre-clinical development of a PSMA targeted minibody, IR800-IAB2M and its first-in-man utilisation for the intraoperative detection of prostate cancer tissue in lymph nodes and extra-prostatic tissues with favourable outcomes. A larger scale evaluation of the reagent is underway using a randomised design to investigate the benefits of real-time intraoperative fluorescent imaging during RARP in achieving complete and optimal tissue excision for improved oncological and functional outcomes in men with prostate cancer. Findings from our study and others should pave the way for a systematic and simultaneous evaluation of multiple molecularly targeted agents using the most cutting-edge fluorescence platforms integrated in robot-assisted surgical equipment, in order to reach consensus and change future practice based on robust evidence.

### Supplementary information

Below is the link to the electronic supplementary material.

Supplementary file1 (DOCX 9218 KB)

## Data Availability

The datasets generated during and/or analysed during the current study are available from the corresponding author on reasonable request.

## Declarations

### IRAS: 234170

The animal facilities and all experiments were approved according to approved UCLA’s Chancellor’s Animal Research Committee (ARC) protocol.
