# Supplementary material for: First-in-man study of the PSMA Minibody IR800-IAB2M for molecularly targeted intraoperative fluorescence guidance during radical prostatectomy
Source: Eur J Nucl Med Mol Imaging. 2024 Jun 10;51(10):3009–25. doi: 10.1007/s00259-024-06713-x (PMC11300503; doi:10.1007/s00259-024-06713-x)
Supplement: Supplementary file 1 — Supplementary file1 (DOCX 9218 KB) [file 259_2024_6713_MOESM1_ESM.docx]

**First-in-man study of the PSMA Minibody IR800-IAB2M for molecularly targeted intraoperative fluorescence guidance during radical prostatectomy**

Freddie C Hamdy*^1,2#^, Alastair Lamb^1,2#^, Iain DC Tullis^3#^, Clare Verrill^1,2^, Ines Rombach^4^, Srinivasa R Rao^1^, Richard Colling^2^ , Paul R Barber^5#^, Davide Volpi^3^, Luis Barbera-Martin^2^, J Francisco Lopez^1,2^, Altan Omer^2^, Aimi Hewitt^1^, Shelagh Lovell^1,2^, Jane Niederer^1,2^, Adam Lambert^1^, Joke Snoeck^1^, Claire Thomson^1^, Tom Leslie^2^, Richard R Bryant^1,2^, Alessandro Mascioni^6^, Fang Jia^6^, Michael Torgov^6^, Ian Wilson^6^, Anna M Wu^7^, Tove Olafsen^7^, Borivoj Vojnovic^3#^

1. Nuffield Department of Surgical Sciences, University of Oxford, UK
2. Oxford University Hospitals NHS Trust, Oxford, UK
3. Department of Oncology, University of Oxford, UK
4. Oxford Clinical Trials Research Unit and Centre for Statistics in Medicine, Nuffield Department of Orthopaedics, Rheumatology and Musculoskeletal Sciences, University of Oxford, UK
5. Comprehensive Cancer Centre, School of Cancer & Pharmaceutical Sciences, King’s College London, London, UK
6. ImaginAb, Inc., Inglewood, CA, USA
7. Beckman Research Institute, City of Hope, Duarte, CA 91010, USA

^#^ These authors provided equal contributions

# S1. Minibody

IAB2M is an 80 kDa molecular-weight minibody with an affinity of 0.08 nM that targets the extracellular domain of PSMA. The anti-PSMA minibody (Mb), IAB2M, was conjugated with the NIR dye, IRDye800CW-NHS ester (Li-cor Biotechnology, Lincoln, NE) on lysine residues in 0.1 Na Borate buffer pH 8.5 using ≥3 molar excess of dye for 2 hours at room temperature to produce the optical imaging agent IR800-IAB2M. The resulting IR800-conjugated IAB2M was formulated in 20 mM Histidine (pH 6.6), 50 mM NaCl and 5% Sucrose. Formulated IR800-IAB2M was analysed for aggregates by size exclusion high pressure liquid chromatography (SE-HPLC) using a Yarra SEC-2000 column with a flow rate of 0.5 mL/min and 0.1 M Sodium phosphate, pH 6.8, as running buffer. Purity and size of the conjugated products were evaluated by sodium dodecyl sulphate–polyacrylamide gel electrophoresis (SDS-PAGE) under both reducing and non-reducing conditions and binding to PSMA expressing cells.

# S2. Cell lines

CWR22Rv1, C4-2, LAPC-4, PC3 human prostate cancer cell lines were obtained from the American Tissue Culture Collection (ATCC, Manassas, VA, USA). The Cal29 human bladder cancer cell line was obtained from DSMZ (Braunschweig, Germany) and the human renal carcinoma RCC4 cells were purchased from ECACC (Salisbury, UK). CWR22Rv1 and C4-2 cell lines were maintained in RPMI-1640, PC3 cells in F-12K media Cal29 and RCC4 cell lines in DMEM (Mediatech, Manassas, VA). The media for all cell lines were supplemented with 10% fetal bovine serum (FBS; Biowest, Kansas City, MO, USA).

# S3. Binding of IR800-IAB2M to PSMA expressing cell lines

The binding properties of IR800-IAB2M to cell surface PSMA was evaluated by FACS analysis and compared to both unconjugated IAB2M (Control) and deferrioxamine (Df)-conjugated IAB2M (Df-IAB2M; Control). Both C4-2 XCL (a subline of the original C4-2 cell line) prostate cancer cell line that expresses approximately 2.2 x 10^5^ PSMA molecules per cell and CWR22Rv1 human prostate carcinoma cells expressing low to moderate levels of endogenous PSMA (approximately 9 x 10^3^ per cell using QIFIKIT from DAKO, Glostrup, Denmark) were used. C4-2 XCL cells were stained after incubating with Control IAB2M or IR800-IAB2M having a Fluorophore to Mb Ratio (FMR) of 1.8. CWR22Rv1 cells were stained after incubating with Control Df-conjugated IAB2M or IR800-IAB2M having an FMR of 1.0 and 2.2. Starting at a concentration of 1.67 µg/mL (22 nM), minibodies were serially diluted 1:3, incubated with cells and detected by Goat Anti-Human IgG (Fcγ Fragment Specific) secondary antibody (Jackson ImmunoResearch Labs, West Grove, PA, USA). Cells were fixed with 1% paraformaldehyde solution following staining. Data for 1 x10^5^ stained cells per point was acquired on the Attune® Cytometer (Thermo Fischer Inc, Waltham, Mass. USA) and analysis performed with 10,000 events/point. EC_50_ values were generated with Graphpad Prism 6 for Windows (GraphPad Software, La Jolla, CA, USA) using four parameter dose response curve analysis.

# S4. Binding specificity of IR800-IAB2M

The *in vitro* specificity of IR800-IAB2M was tested on different cell lines that do and do not express PSMA. 2 x 10^4^ cells per well were plated in a multi-well glass-bottom plate. One day later the medium was replaced with 100 μL of medium containing 2 μg/mL of IR800-IAB2M. This concentration was selected as it represents the level of drug that is expected to be achieved during clinical imaging. The cells were imaged (Supplementary Figure 3A) for uptake of NIR dye using an Indocyanine Green (ICG) filter set at an excitation wavelength of 785 nm.

Specific binding was also assessed by competitive FACS binding assay using the C4-2 XCL prostate cancer cell line that expresses approximately 220,000 PSMA molecules per cell. A constant amount of IAB2M labelled with Alexa Fluor 488 (AF488-IAB2M; 0.1 µg/mL) was competed against increasing concentrations of IAB2M and IR800-IAB2M. Data was acquired on the Attune® Acoustic Focusing Cytometer (Applied Biosystems) and the mean fluorescent intensity (MFI) values were plotted against the concentration for each sample. (Supplementary Figure 2B). Best fit binding curves and IC_50_ values were generated with four-parameter logistic nonlinear regression using GraphPad Prism 6 for Windows.

# S5. In vitro characterization of IR800-IAB2M

Several batches of IRDye800-IAB2M with different Flurophore-to-Minibody-Ratios (FMR) were generated. An FMR of ≤2 was considered optimal for IAB2M. Size exclusion chromatography showed that >96 % of the IR800-IAB2M with an FMR of 1.8 and at concentrations of 5 and 7 mg/ml, eluted almost at the same time as the unconjugated IAB2M (Supplementary Figure 1A). SDS-PAGE showed that IR800-IAB2M mainly migrated as an 80 kDa fragment under non reducing conditions, that was reduced to 40 kDa consistent with the IAB2M (Supplementary Figure 1B).

**Supplementary Figure 1.** IRdye800 excitation (dotted black line) and emission (solid black line) plotted alongside excitation wavelengths and emission filter passbands for the clinical system used (solid red line and light grey shading respectively and the optical system in the da Vinci Xi robot with Firefly attachment (dotted red line and darker grey shading respectively).

**Supplementary Figure 1.** In vitro characterization of IR800-IAB2M. A) Size exclusion chromatography of IR800-IAB2M with FMR 1.8 and IAB2M. B) SDS-PAGE of IR800-IAB2M with FMR 1.8 and IAB2M under non-reducing (N.R.) and reducing (Red.) conditions. Molecular weight standard is shown.


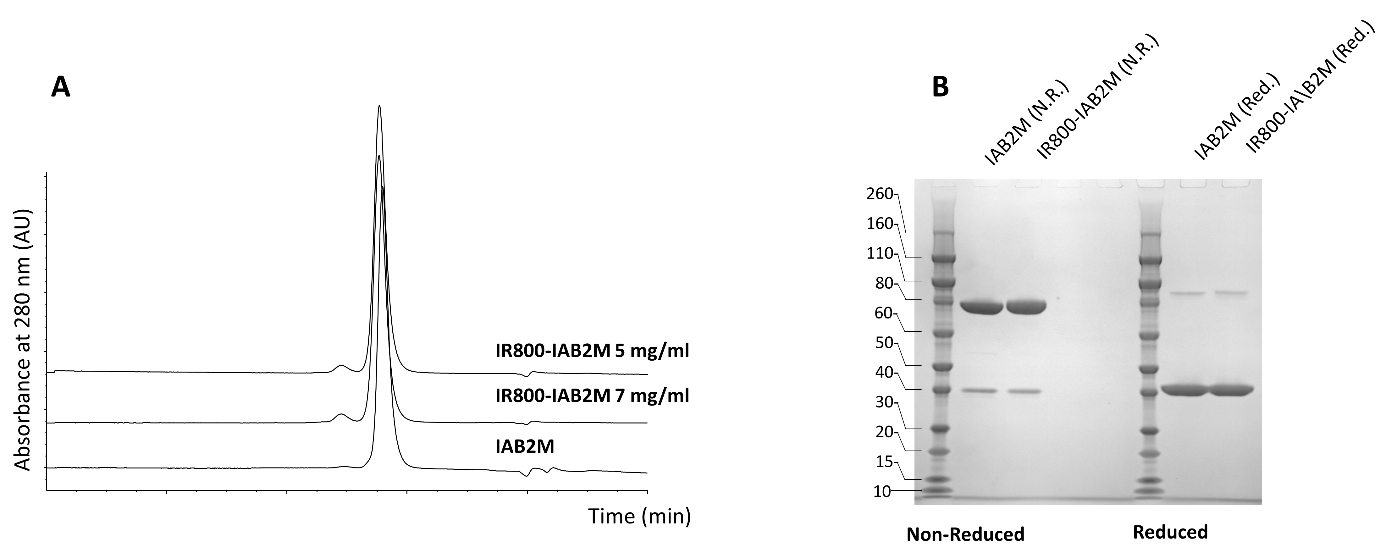


Binding of Df-IAB2M (Control) and IR800-IAB2M to PSMA was evaluated by Fluorescence-Activated Cell Sorting (FACS) using both C4-2 XCL and CWR22Rv1 human prostate carcinoma cell lines. Binding of IR800-IAB2M with an FMR of 1.8 to C4-2 XCL cells was compared to unconjugated IAB2M (Supplementary Figure 2A). For this cell line the calculated EC_50_ was 0.142 nM for IRDye800-IAB2M versus 0.184 nM for IAB2M. The binding of IR800-IAB2M conjugated with two FMR was evaluated with the CW22Rv1 cell line and demonstrated similar binding affinity for PSMA as Df-IAB2M. In this cell line the EC_50_ values were determined to be 0.1, 0.095 and 0.06 nM for IR800-IAB2M (FMR = 1.0), IRDye800-IAB2M (FMR = 2.2) and Df-IAB2M, respectively (Supplementary Figure 2B). No binding to PSMA negative PC3 cells was observed at the highest concentrations tested (data not shown).

**Supplementary Figure 2.** In vitro characterization of IR800-IAB2M. A) Binding of IR800-IAB2M with FMR 1.8 and IAB2M to C4-2 XCL. The EC_50_ values are shown in the table the graph. B) Binding of IR800-IAB2M with FMR 1.0 and 2.2 and Df-IAB2M to CWR22Rv1. The EC50 values are shown in the tables below the graph.


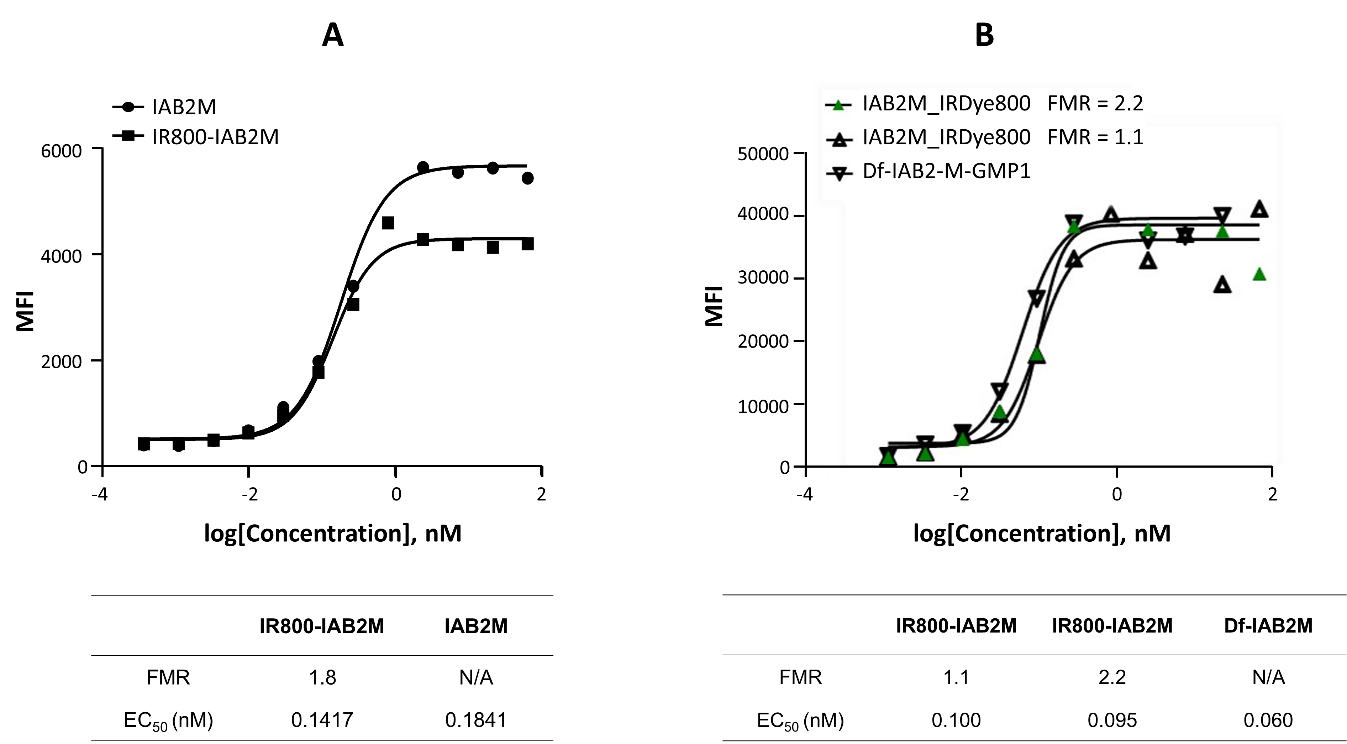


The specificity of IR800-IAB2M was confirmed by incubating IRDye800-IAB2M with PSMA-expressing and negative control cell lines. A strong NIR fluorescence signal was obtained with the PSMA expressing CW22Rv1 and LNCaP prostate tumour cell lines, whereas the non-PSMA expressing Cal29 bladder and RCC4 kidney tumour cell lines exhibited ~100-fold lower fluorescence signal (Supplementary Figure 3A).


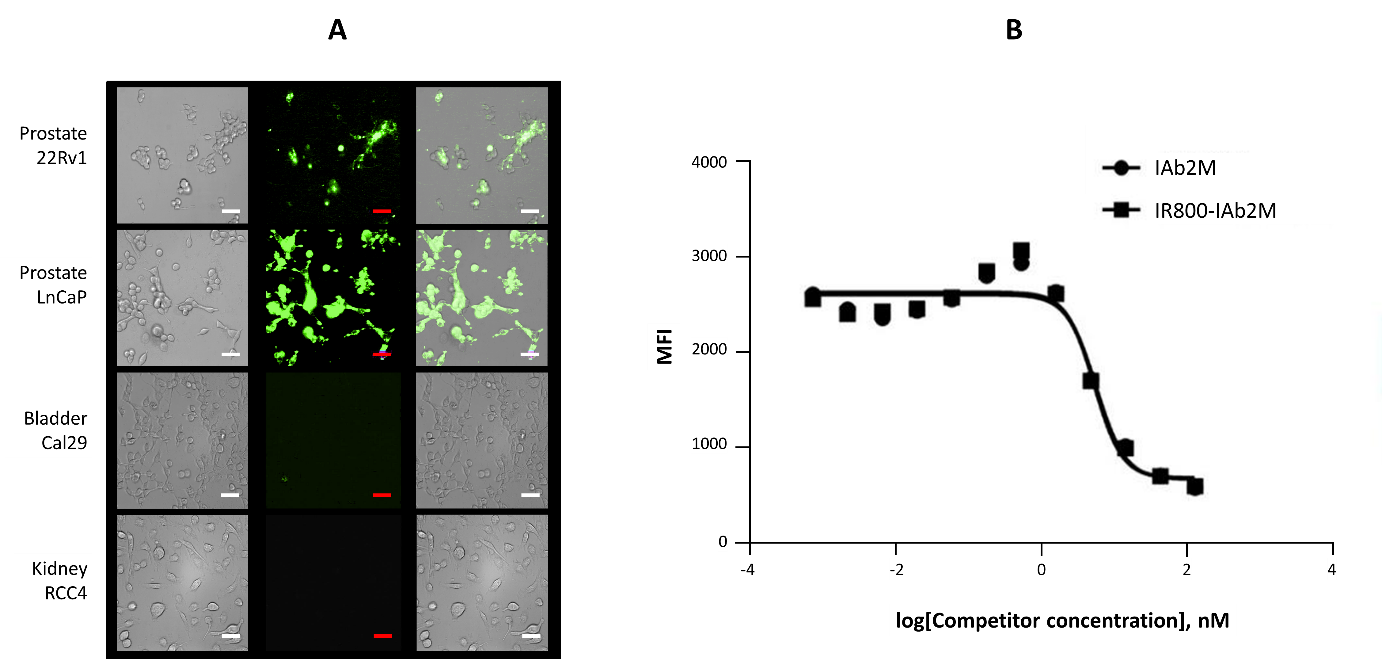


**Supplementary Figure 3.** In vitro characterization of IR800-IAB2M. A) Specificity of IR800-IAB2M for binding and uptake into cells expressing PSMA. Left panels show white light phase contrast imaging; Middle panels show NIR fluorescence imaging; Right panels show overlays of NIR fluorescence on phase contrast images for the respective cell lines. B) Competitive binding of IAB2M and IR800-IAB2M to PSMA on C4-2 XCL prostate cancer cells.

In addition, both unconjugated IAB2M and conjugated IRDye800-IAB2M demonstrated similar competition for binding to PSMA expressed on the surface of C4-2 XCL prostate cancer cells with IC_50_ values of 5.41 and 5.71 nM, respectively (Supplementary Figure 3B).

# S6. In vivo characterization of IR800-IAB2M

Initial *in vivo* preclinical evaluation of IR800-IAB2M was performed at the Preclinical Imaging Technology Center (PITC) of The Crump Institute for Molecular Imaging (University of California, Los Angeles (UCLA), CA) using the IVIS Lumina II (Perkin Elmer Inc, Waltham, Mass. USA). Images were analysed by drawing regions of interest (ROIs) over the tumour and the contralateral side (background) to determine the tumour uptake and tumour to background ratio (TBR). Animals bearing both low to moderate and high PSMA expressing xenografts as well as PSMA-negative tumours were imaged in IVIS Lumina II following intravenous administration of IR800-IAB2M. Mice bearing CW22Rv1 xenografts are shown in Supplementary Figure 4. In these animals, high background with a fluorescent signal in the tumour was visible at 3 hours post injection. As the minibody cleared from the blood, image contrast improved with a strong signal detected in both the tumour and the liver.

Post-mortem images revealed that the fluorescent signal was limited to the tumour, liver and kidneys in the same mouse (Supplementary Figure 4B). The liver signal was expected as this is the major organ for minibody clearance. Image analysis showed substantial reduction in liver and kidney uptakes from 48 to 72 hours, whereas the tumour uptake remained almost the same (Supplementary Figure 4C). This trend shows that tumour-to-background ratios improved over time and that the optimal time for image contrast and sensitivity, in mice at least, after administration of IR800-IAB2M ranged from 24 h to 48 h in mice.

**Supplementary Figure 4.** Optical imaging of with IVIS Lumina II. (A) In vivo serial optical scans of a representative nude mouse bearing human CWR22Rv1 prostate cancer xenografts indicated by the yellow arrow at 3, 24, 48 and 72 hours post injection of IR800-IAB2M. Each image is scaled differently as shown by the radiant efficiency values next to each image. (B) Ex vivo image of harvested organs at 72 hours post-injection and the corresponding radiant efficiency. (C) Mean (±SEM) fluorescent intensity in tumour, liver, kidney and muscle in 2 mice sacrificed at 48 and at 72 hours post-injection. The TBRs, in this instance using muscle as the ‘background’, for both times are shown in the plot in the inset.


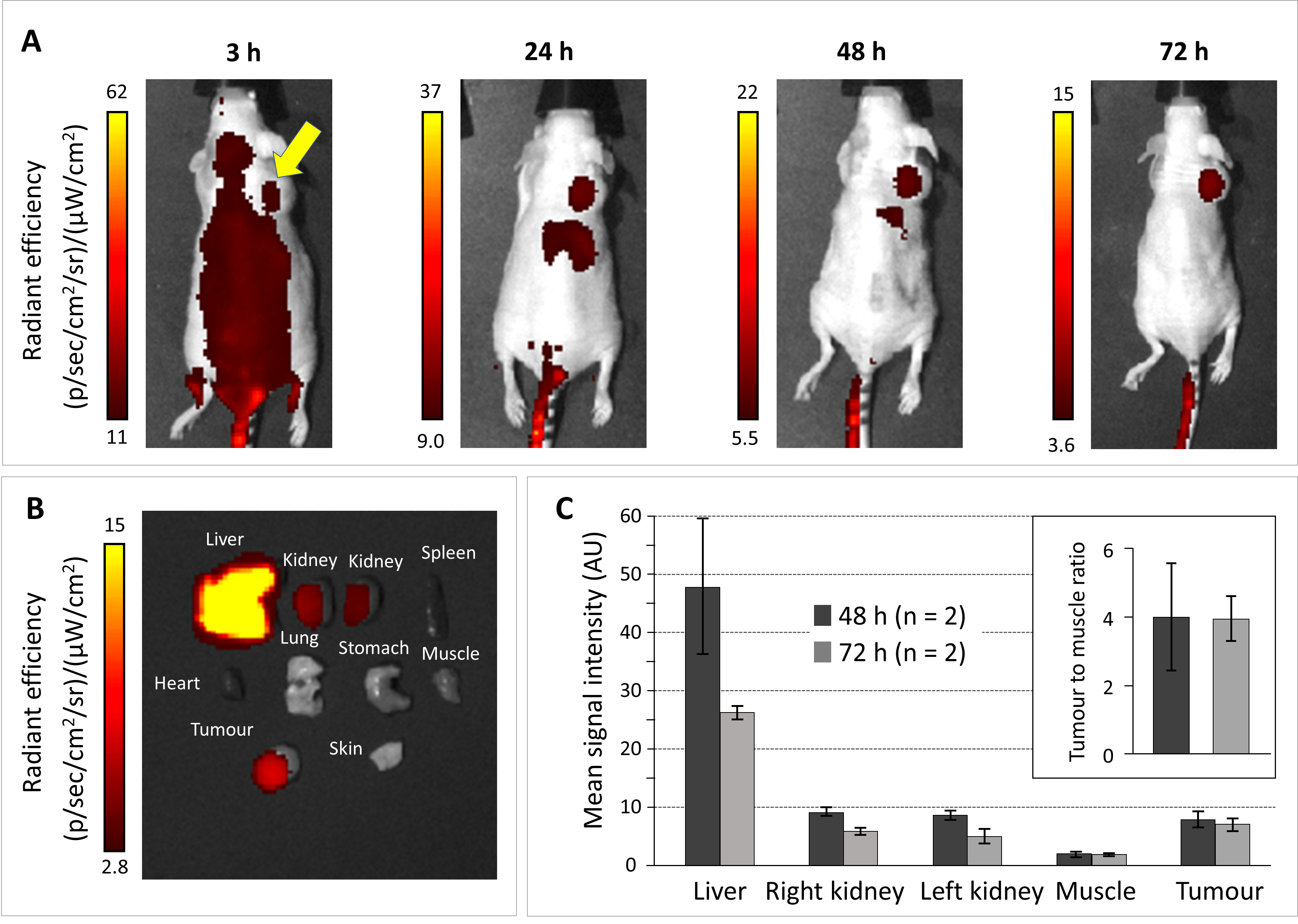


#
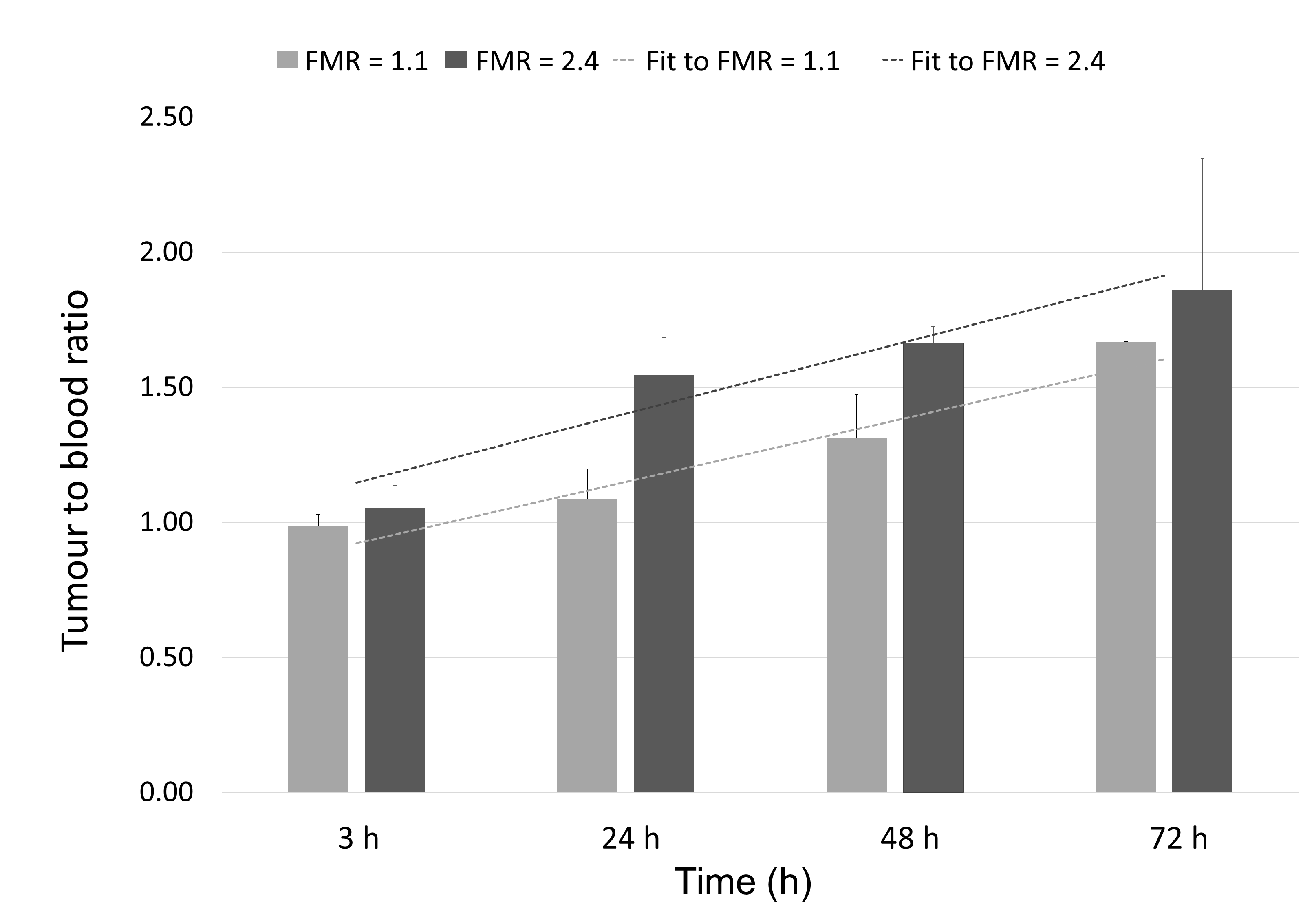
S6. Tumour to Blood ratios in mice

Image contrast represented by the tumour to blood ratios (TBdR) improved over time while the overall tumour uptake diminished for both FMRs of 1.1 and 2.4 (Supplementary Figure 5). The trendline shows that tumour-to-blood ratios improved over time for both constructs. When considered together the results from this experiment demonstrate that prostate tumours expressing a relatively low level of PSMA can be detected by fluorescent imaging after administration of IR800-IAB2M with two different ratios of NIR fluorophore attached. The optimal time for image contrast and sensitivity ranged from 24 h to 48 h in mice.

**Supplementary Figure 5.** In vivo fluorescence in mice: Tumour to blood ratios for different FMRs at increasing times post injection of marker.

# S7. Imaging devices

## S7.1. Pre-clinical imager details

For preclinical imaging, we used a 1/3" interline CCD PAL Colour analogue camera (XC-505P, Sony Corporation, Tokyo, Japan) with its internal NIR filter removed and fitted with a 25 mm focal length vis.-NIR C-series lens, #67-715, (Edmund Optics Ltd, York, UK) and a fluorescence excitation blocking notch filter (NF03-785E-25, Semrock, Rochester, NY, USA). A LZ1-00CW05 Light emitting diode (LED) (LED Engin Inc, CA, USA), and associated optics provides white light illumination. Fluorescence excitation is provided by K78S09F-1.60W (BWT, Beijing, China) solid-state lasers, fibre coupled to 105 μm core fibres. Three such fibre lasers, providing an optical power of 1.2 W each, are coupled into a 1 mm diameter bundle and more lasers could be used if required. A 650 nm corner wavelength dichromatic mirror (Semrock, Rochester, NY, USA) combines collimated LED white light and collimated bundle output light and launched into a liquid light guide (LLG20.530, Lumatec GmbH, Oberhaching, Germany) that provides widefield illumination. The white light illumination is controlled by a linear automatic gain control circuit using image intensity as a feedback signal and compared with a user set-point. The fluorescence excitation can be added as required to result in combined white-light and fluorescence imaging [1, 2]. The gamma-compressed (γ = 0.45) PAL output is digitised with a VRmAVC-2 converter (VRmagic Imaging Haag-Streit GmbH, Mannheim, Germany) and stored in a Windows PC.

## S7.2. Clinical imager details

Clinical imaging was performed with a Sony Super HAD CCD II 1/3" Sensor (Sony ICX693) was used in a Blackfly USB 3.0 camera, (BFLY-U3-13S2M-CS, FLIR Systems UK, West Malling, UK) with the camera’s internal NIR-blocking filter removed.

Fluorescence excitation is provided by a custom fibre-coupled ‘half-bar’ solid-state laser (Coherent Dilas Mainz, Germany) fitted with a volume Bragg grating and delivering >10 W at 779.6 nm. White light illumination is provided by Luminus (Sunnyvale, CA, USA) high power light-emitting diode and is combined with the collimated laser output using a dichromatic reflector (Semrock, Rochester, NY, USA) before launching into the same liquid light guide as described previously.

The LED output is sampled with a 1 mm polymer optical fibre to enable it to be placed in a logarithmic feedback loop that controls the LED intensity so as to maintain the output image intensity to any desired user-set level of illumination. It is this feature that allows us to exploit the wide dynamic range provided by the camera allowing us to increase the programmable integration time up to 1 second and exploit camera gains of up to 24 dB (i.e. x16). Moreover, the camera allows pixel binning and we used binning at up to 2 x 2 pixels as required to increase sensitivity at the expense of spatial resolution.

The camera’s USB3 output was acquired via the manufacturer-supplied SDK. The main executable program on the video processor is implemented in the C programming language using Glib libraries and compiled using Gnu tools ([gcc.gnu.org/](https://gcc.gnu.org/)). The video processor code executes on the quad-core Intel® Core^TM^ i7 Haswell, Commell type LE-37C single board host computer (Taiwan Commate Computer Inc., New Taipei City, Taiwan), running Ubuntu Linux 12·04 LTS 64-bit, with 8 GB RAM. Control communications associated with the in-house developed laser and LED drivers as well as associated thermoelectric coolers was performed via local I^2^C and SPI channels, along with other serial communications. Image and acquisition metadata were displayed on a medical-grade monitor and stored on a removable solid-state hard disc. Although the camera output provides 3 x 8 bit RGB images, its internal analogue-digital converter operates at up to 3 x 12 bits and the output look-up table can be set to any desired characteristic.

**Supplementary Figure 6.** Block diagram of the clinical imager. The light outputs of the white light LED (Light Emitting Diode) and NIR (Near-Infra-Red) laser sources is controlled by in-house developed drivers using switch-mode converters, driven by digital to analogue converters (DACs). These optical outputs are combined with a dichromatic reflector and they feed a flexible light guide connected to the laparoscope. The laparoscope output port illuminates a camera through a notch filter (to reject excitation light) and a focus-programmable lens. A quad-core processor performs all imaging processing and control functions and is able to feed two display monitors and a hard disk for video storage. The instrument is controlled through a touch screen.


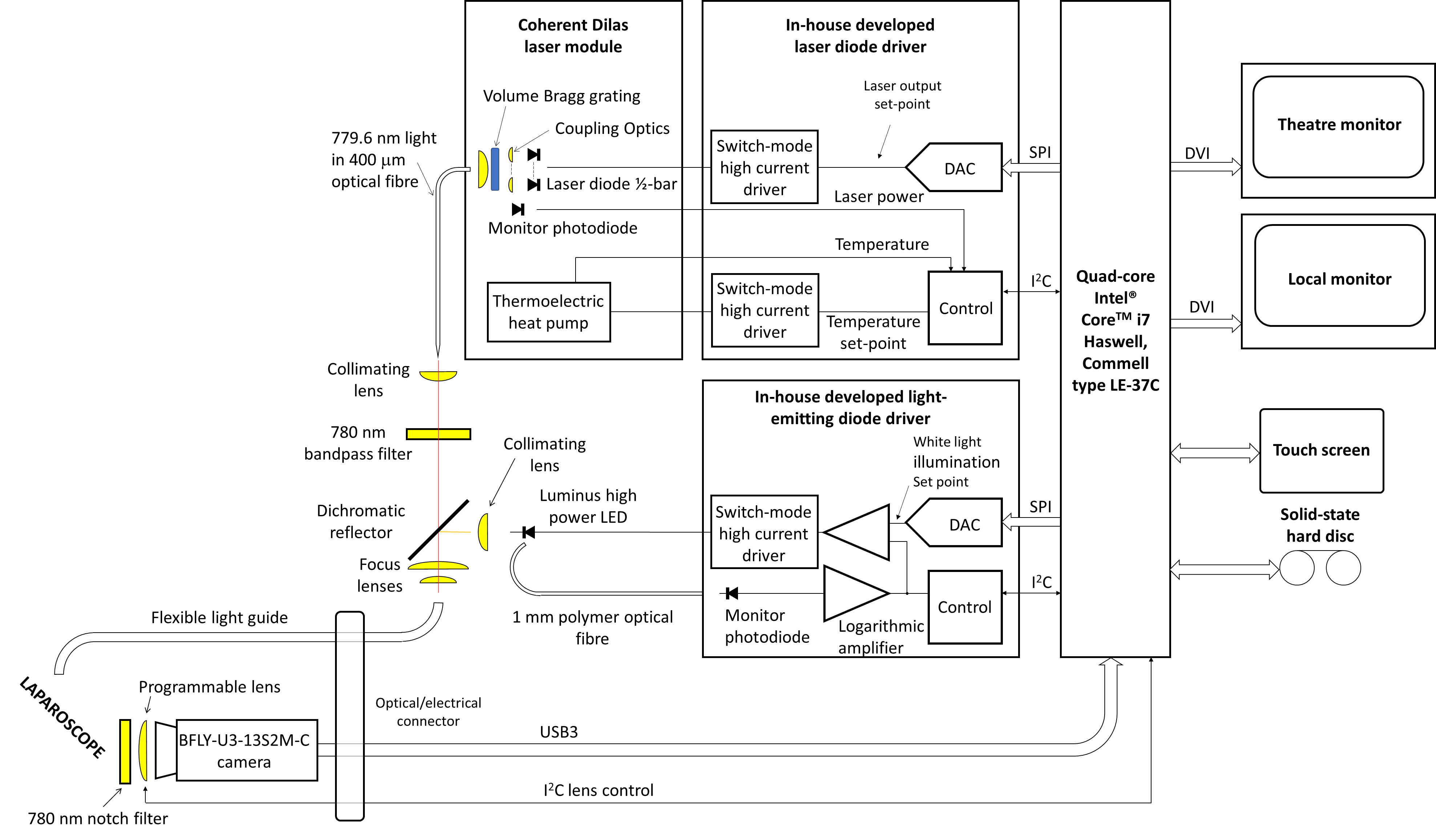
**S8. Fluorescence Spectra and compatibility with other clinical imaging devices**

Most clinical fluorescence detection devices are geared towards indocyanine green (ICG) imaging. Although ICG can also be excited at wavelengths ~780 nm [3], most commercial devices use 805 nm for excitation as this is somewhat more optimal and laser sources are readily available at this wavelength at low cost. ICG has a wide excitation spectrum as well as a large Stokes shift, emitting at well above 850 nm. The fluorophore used in this work, IRDye800, is ideally excited at 775 nm. Laser sources at 775 nm are not readily available and we chose to use an excitation wavelength of just under 780 nm: this reduces the fluorescence yield by ~5%. The excitation and emission spectra for IRDye800, along with excitation and emission wavelength bands used by our clinical system and that of the DA Vinci Xi /Firefly system are shown in Supplementary Figure 7.

It would be expected that the excitation sensitivity of the Xi would be ~22.4% of that of our device when. Similarly, if only the emission sensitivity is considered, the change in wavelengths would result in ~31% of the emission sensitivity of our device. Overall, for comparable excitation power densities, camera gains and integration times, it would be expected that only ~7% of the sensitivity would be available with the Xi device: under similar excitation conditions our device should thus be ~14x more sensitive. However, it is also likely that the optical losses in our device are also higher (use of a light guide, programmable lens optics etc.) and it would be reasonable to expect the relative sensitivity is reduced somewhat to ~10x. Furthermore, the imaged area with the Firefly is approximately 1.5x larger, so the more realistic figure for the underlying improvement in sensitivity is ~x6.7. This underlying increase can be determined with a fluorescence phantom, as shown in Supplementary Figure 8.

**Supplementary Figure 7.** IRdye800 excitation (dotted black line) and emission (solid black line) plotted alongside excitation wavelengths and emission filter passbands for the clinical system used (solid red line and light grey shading respectively and the optical system in the da Vinci Xi robot with Firefly attachment (dotted red line and darker grey shading respectively).


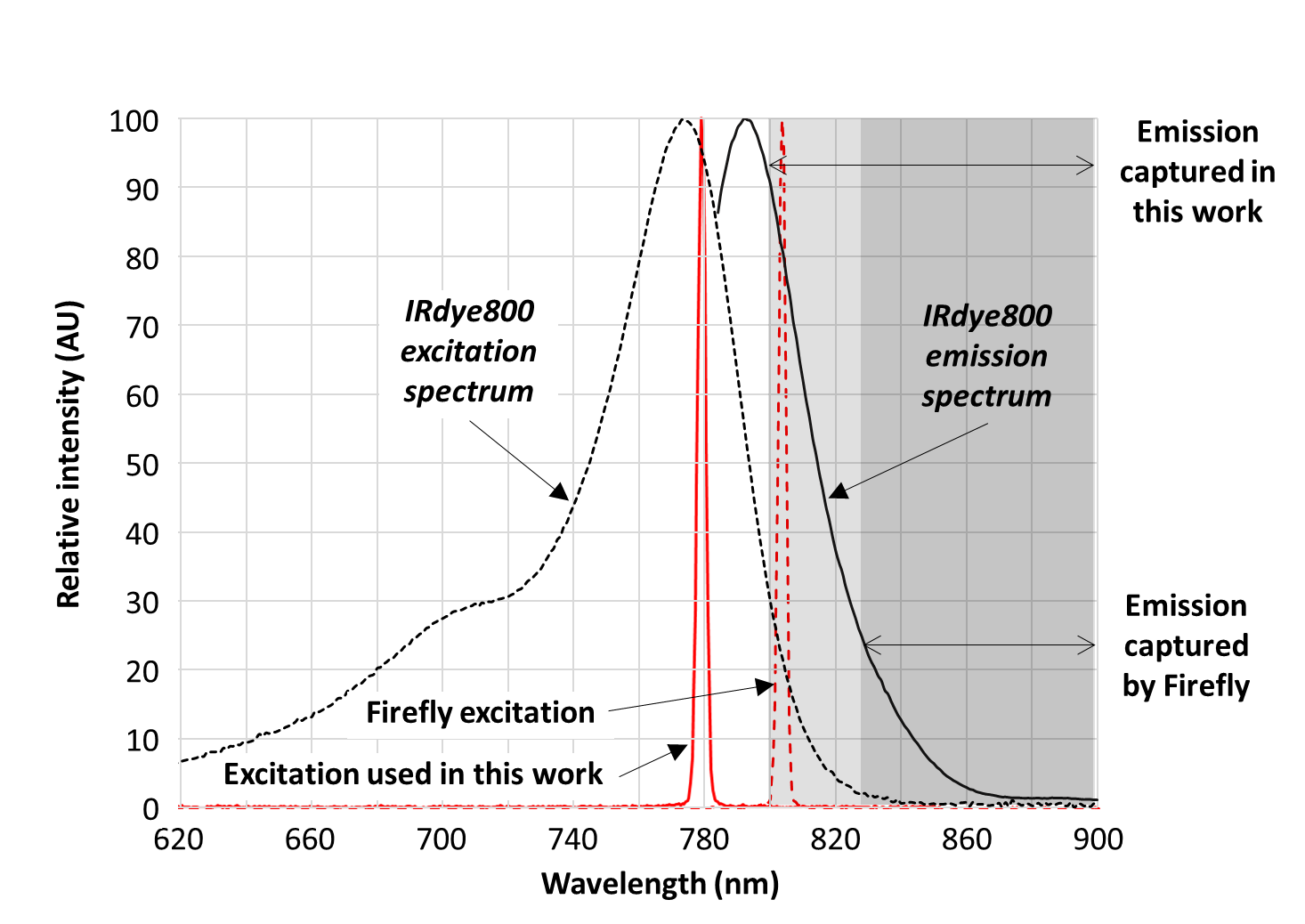


A further important issue is that of fluorescence image overlays used in the Da Vinci, as well as in other commercial devices. A wide range of approaches is used [4] and it is not clear to us whether thresholding or a non-linear look-up table is applied in the Da Vinci. It is noted that conventionally FGS is used purely for visualising the location of the fluorescence in the imaged field and not for intensity measurement purposes. In our case, particularly when using very low marker concentrations, it has been found that intensity measurements are desirable in order to derive TBRs. We thus linearize image data prior to fluorescence intensity measurement.

In Supplementary Figure 8, the Firefly and our system can be inter-compared by reducing the excitation intensity of our system so as to obtain comparable displayed fluorescence intensities. In addition, the intensity of the green ‘overlay’ component used in the Firefly was determined through image analysis and interpolation. This determination can only be considered as very approximate due to the limited dynamic range present in the image. Once the difference in excitation intensities, system gains and overlays are considered, the underlying sensitivity improvement is of our system x5.2 over that of the Firefly. This figure is close to the x6.7 derived from spectrum estimation. Inevitably compression algorithms contribute to this difference.

**Supplementary Figure 8.** Comparison of portions of images of a IR800-IAB2M phantom acquired with the Da Vinci Xi/Firefly system, (Panel A), with those acquired with our custom imaging system (Panel B), with the fluorescence excitation intensity adjusted to provide comparable image intensities. Note that the Firefly image appears brighter due to the use of a transparent overlay. The image in panel A is acquired at a 13x larger excitation power density, while the image in panel B is acquired at a 4x longer integration time. The Intensity of the underlying ‘green’ component in image A is 1.6x lower than that of the image in panel B. The underlying sensitivity of the custom system is thus 5.2x that of the Firefly.


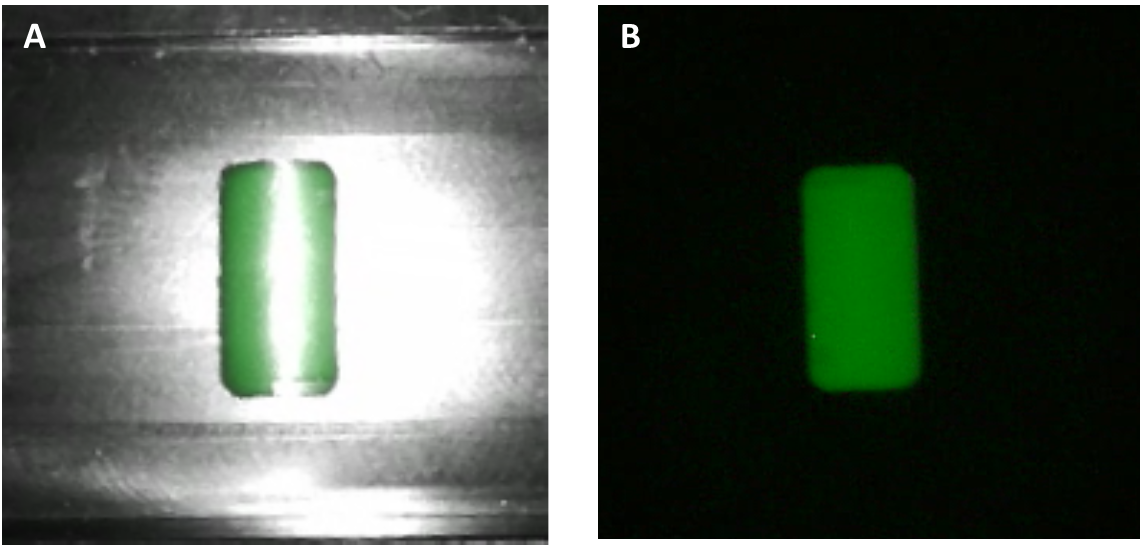


It is noted that it was not possible to turn off the non-fluorescence illumination (blue light) on the Da Vinci/Firefly available to us. Hence the monochrome white light reflection artefacts on the left image of Supplementary Figure 8. It is also noted that our system allows a very wide variation in camera integration times and gains. We routinely used ~8-fold greater camera sensitivity than those used during the acquisition presented in Supplementary Figure 8 when performing imaging at low marker concentrations. This would suggest that our system, under these conditions, could be ~40-fold higher than that of the Da Vinci instrument available to us. Furthermore, we can readily increase that to >>100x through the use of pixel binning and longer integration times. Of course, such drastic increases in sensitivity are inevitably accompanied by random image noise, but since a large number of pixels (typically >100-<5000) are usually used for determining TBRs, even TBR value close to unity can be readily determined.

There are no fundamental reasons why the excitation and emission wavelengths could not be matched to the IRDye800 fluorophore in other commercial systems, and indeed in the Firefly. However, the differences in the way the fluorescence information is processed, at least in the current versions of the Firefly, makes it difficult to perform direct comparisons.

# S9. Fluorescence from urine

Measurements of fluorescence intensity from urine from 13 out of the 24 patients show a good correlation between administered dose and fluorescence intensity as shown in Panel A of Supplementary Figure 9. Measurement sensitivity and sample volume were maintained constant throughout the acquisition of individual kinetics. These were fitted with an exponential decay function and the time zero extrapolated intensity determined. This was then used to normalise the data presented in Supplementary Figure 9, which also shows the exponential fit that includes all the data points. Inter- and intra-patient variability is the reason why this overall fit does not start at 100 AUs, the intensity to which individual fits were normalised to. These data suggest that the fluorescence clears with a 1/e time of ~5-6 days and that it is has returned to close to baseline levels within 20 days at most.


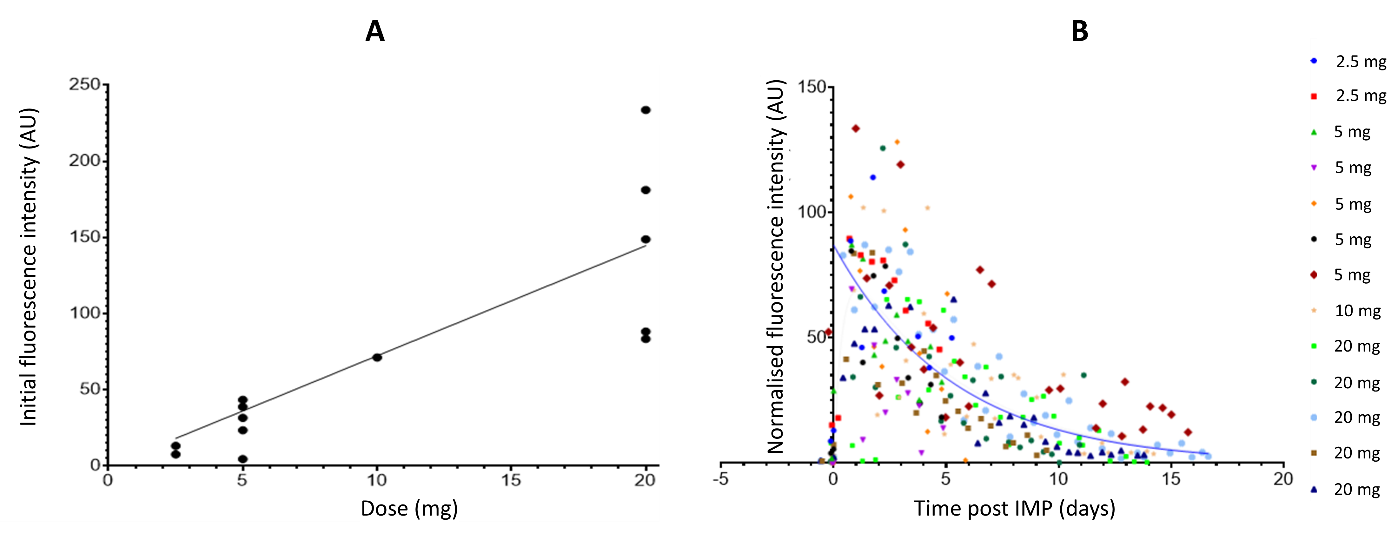


**Supplementary Figure 9.** Panel A: Urine fluorescence intensity, extrapolated to time of injection, as a function targeting agent dose. Panel B: Normalised fluorescence intensity of urine as a function of time for different doses across the 13 patients studied.

# S10. Supplementary references

Volpi D, Tullis IDC, Laios A, Pathiraja PNJ, Haldar K, Ahmed AA, Vojnovic B. “A novel multiwavelength fluorescence image-guided surgery imaging system” (2014) Proceedings of SPIE, 8935, 89350G-89350G-9; doi: [10.1117/12.2038377](https://doi.org/10.1117/12.2038377).

2 Laios A, Volpi D, Tullis, IDC, Woodward M, Kennedy S, Pathiraja P, Haldar K, Vojnovic B, Ahmed, AA. “A prospective pilot study of detection of sentinel lymph nodes in gynaecological cancers using a novel near infrared fluorescence imaging system”, (2015) BMC Research Notes **8**(608), 1-9; doi: [10.1186/s13104-015-1576-z](https://doi.org/10.1186/s13104-015-1576-z).

3 Ochoa MI, Ruiz A, LaRochelle E, Reed M, Berber E, Poultsides G, and Pogue BW. Assessment of open-field fluorescence guided surgery systems: implementing a standardized method for characterization and comparison J Biomed Opt. 2023 Sep; 28(9): 096007. PMCID: PMC10513724, PMID: 37745774; doi: [10.1117/1.JBO.28.9.096007](https://doi.org/10.1117/1.JBO.28.9.096007).

# 4 Elliott, JT, Dsouza AV, Davis SC, Olson JD, Paulsen KD, Roberts, DW and Pogue BW. Review of fluorescence guided surgery visualization and overlay techniques. Biomed Opt Express. 2015 Oct 1; 6(10):3765–3782; doi: [10.1364/BOE.6.003765](https://doi.org/10.1364/BOE.6.003765) PMID: 26504628
